# Tunable Layered (Na,Mn)V_8_O_20_·*n*H_2_O Cathode Material for High‐Performance Aqueous Zinc Ion Batteries

**DOI:** 10.1002/advs.202000083

**Published:** 2020-05-28

**Authors:** Min Du, Chaofeng Liu, Feng Zhang, Wentao Dong, Xiaofei Zhang, Yuanhua Sang, Jian‐Jun Wang, Yu‐Guo Guo, Hong Liu, Shuhua Wang

**Affiliations:** ^1^ State Key Laboratory of Crystal Materials Shandong University Jinan 250100 P. R. China; ^2^ Department of Materials Science and Engineering University of Washington Seattle WA 98195 USA; ^3^ CAS Key Laboratory of Molecular Nanostructure and Nanotechnology CAS Research/Education Center for Excellence in Molecular Sciences Beijing National Laboratory for Molecular Sciences (BNLMS) Institute of Chemistry Chinese Academy of Sciences (CAS) Beijing 100190 P. R. China; ^4^ Institute for Advanced Interdisciplinary Research (iAIR) University of Jinan Jinan 250022 P. R. China

**Keywords:** aqueous zinc‐ion batteries, dissolution, doped, energy storage mechanisms, transition metals

## Abstract

Rechargeable aqueous zinc‐ion batteries (ZIBs) show promise for use in energy storage. However, the development of ZIBs has been plagued by the limited cathode candidates, which usually show low capacity or poor cycling performance. Here, a reversible Zn//(Na,Mn)V_8_O_20_·*n*H_2_O system is reported, the introduction of manganese (Mn) ions in NaV_8_O_20_ to form (Na,Mn)V_8_O_20_ exhibits an outstanding electrochemical performance with a capacity of 377 mA h g^−1^ at a current density of 0.1 A g^−1^. Through experimental and theoretical results, it is discovered that the outstanding performance of (Na,Mn)V_8_O_20_·*n*H_2_O is ascribed to the Mn^2+^/Mn^3+^‐induced high electrical conductivity and Na^+^‐induced fast migration of Zn^2+^. Other cathode materials derived from (Na,Mn)V_8_O_20_·*n*H_2_O by substituting Mn with Fe, Co, Ni, Ca, and K are explored to confirm the unique advantages of transition metal ions. With an increase in Mn content in NaV_8_O_20_, (Na_0.33_,Mn_0.65_)V_8_O_20_ ·*n*H_2_O can deliver a reversible capacity of 150 mA h g^−1^ and a capacity retention of 99% after 1000 cycles, which may open new opportunities for the development of high‐performance aqueous ZIBs.

## Graphical abstract

Mn‐doped NaV_8_O_20_ is synthesized via a one‐step hydrothermal reaction. The Mott–Schottky plots, Tafel curves and calculation results are given to explain the improved cycling performance of Mn‐doped NaV_8_O_20_. Other cathode materials derived from (Na,Mn)V_8_O_20_·*n*H_2_O by substituting Mn with Fe, Co, Ni, Ca, and K are explored to confirm the unique advantages of transition metal ions in NaV_8_O_20_.

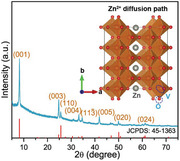

## 1 Introduction

Given the looming energy crisis and environmental pollution, renewable green energy resources such as wind and solar have attracted widespread attention in recent years.^[^ ^1^, ^2^, ^3^, ^4^ ^]^ Lithium‐ion batteries have been widely used in energy storage due to their high energy density.^[^ ^5^, ^6^ ^]^ However, the toxicity and flammability of organic electrolytes cause environmental problems and safety issues. Reliable strategies such as the utilization of solid‐state electrolytes or aqueous electrolytes seem to overcome these dilemma.^[^ ^7^ ^]^ Aqueous rechargeable batteries are competitive alternatives because aqueous electrolytes are characterized by lower cost, higher safety, greater environmental friendliness, and higher ionic conductivity (up to 1 S cm^−1^) than organic electrolytes or solid‐state electrolytes.^[^ ^2^, ^8^, ^9^ ^]^ Aqueous Zn‐ion batteries (ZIBs) using metallic Zn as the anode have attracted surging interest due to their high specific capacity of 819 mA h g^−1^ at a low redox potential (−0.762 V versus the standard hydrogen electrode).^[^ ^2^, ^10^, ^11^, ^12^ ^]^ Unfortunately, exploiting suitable cathode materials with high specific capacity and stable cycling performance is still an urgent task, and it limits the practical and large‐scale applications of ZIBs.

Considerable research efforts have been devoted to exploring cathode materials,^[^ ^13^, ^14^, ^15^, ^16^, ^17^, ^18^, ^19^, ^20^, ^21^, ^22^, ^23^, ^24^, ^25^, ^26^, ^27^, ^28^, ^29^, ^30^, ^31^, ^32^, ^33^ ^]^ such as Mn‐based oxides,^[^ ^13^ ^]^ Prussian blue analogs,^[^ ^14^, ^22^ ^]^ transition metal sulfides,^[^ ^16^ ^]^ and vanadium‐based compounds^[^ ^19^, ^20^, ^21^, ^22^, ^23^, ^24^, ^25^, ^26^, ^27^, ^28^, ^29^, ^30^, ^31^, ^32^, ^33^ ^]^ for aqueous ZIBs. MnO_2_ displays a high operating voltage around 1.3 V but suffers from the irreversible phase transition and Mn cations dissolution during the cycling.^[^ ^13^ ^]^ Prussian blue analogs deliver a low capacity of ≈100 mA h g^−1^ and are hindered by their sluggish kinetics.^[^ ^14^, ^22^ ^]^ Layered vanadium‐based oxides have high capacity exceeding 300 mA h g^−1^ but suffer from structural degradation.^[^ ^20^, ^21^, ^22^, ^23^, ^24^ ^]^ Incorporating metal (Mn, Co, Ni, Fe, Zn, Li, Na) cations and/or molecular water (H_2_O) into V_2_O_5_ has been proven to be an effective method for solving the poor long‐term cycling performance of vanadium‐based oxides.^[^ ^19^, ^20^, ^21^, ^22^, ^23^, ^24^ ^]^ Structural H_2_O in bilayer V_2_O_5_ reduces the electrostatic interactions within the V_2_O_5_ framework, thus effectively promoting Zn^2+^ diffusion.^[^ ^19^ ^]^ Metal ion‐preintercalated V_2_O_5_ usually expands the interlayer spacing more than V_2_O_5_.^[^ ^22^, ^23^ ^]^ The expanded interlayer spacing could provide fast channel for Zn^2+^ diffusion and improve the reaction kinetics, and the chemically connected layers become more robust to alleviate the structural degradation, resulting for a long‐term cycling stability as well as excellent rate capability. Apart from V_2_O_5_ derivatives, other layered vanadium‐based oxides, such as NaV_3_O_8_ composed of V_3_O_8_ layers and inserted sodium ions, delivers a capacity of 380 mA h g^−1^ at a current density of 0.05 A g^−1^.^[^ ^27^ ^]^ However, NaV_3_O_8_ gradually dissolved into the ZnSO_4_ electrolyte and caused a rapid degradation in capacity during cycling. The addition of high concentration Na_2_SO_4_ into the electrolyte can suppress the dissolution equilibrium of Na^+^ from NaV_3_O_8_ because of common‐ion effect. Apart from adding electrolyte additives, exploring other strategies for improving the structural stability of these cathodes during cycling is of essential importance in ZIBs. Until now, many metal ions pre‐intercalated strategies have focused on V_2_O_5_‐based cathodes.^[^ ^20^, ^21^, ^22^, ^23^, ^24^, ^25^, ^26^ ^]^ However, the different role of transition metal cations and alkali metal cations in improving the electrochemical performance of vanadium‐based materials is unclear. New compounds intended as cathode materials for ZIBs could be explored by intercalating transition metal ions such as Mn into NaV_8_O_20_, forming (Na,Mn)V_8_O_20_·*n*H_2_O.

Here we report a hydrated, monovalent and divalent/trivalent cations co‐pre‐inserted V_8_O_20_ nanobelts as cathode materials for ZIBs, and we show strong experimental and theoretical evidences that the outstanding performance of (Na,Mn)V_8_O_20_·*n*H_2_O is ascribed to the Mn^2+^/Mn^3+^‐induced high electrical conductivity and Na^+^‐induced fast migration of Zn^2+^. The Mn ions stabilize the NaV_8_O_20_·*n*H_2_O (NVO) structure during the charge/discharge process. As the positive electrode for aqueous ZIBs, NVO delivers a capacity of 140 mA h g^−1^ and exhibits a capacity retention of 69% after 1000 cycles at 4 A g^−1^, while (Na_0.97_,Mn_0.02_)V_8_O_20_·*n*H_2_O delivers a reversible capacity of 146 mA h g^−1^ and a capacity retention of 88% after 1000 cycles. With an increase in Mn content in NVO, (Na_0.33_,Mn_0.65_)V_8_O_20_·*n*H_2_O can deliver a reversible capacity of 150 mA h g^−1^ and a capacity retention of 99% after 1000 cycles. Other cathode materials that were derived from (Na,Mn)V_8_O_20_·*n*H_2_O by substituting Na with K, Li, and Zn are explored and deliver long‐term cycling stability. Furthermore, derivative cathode materials from (Na,Mn)V_8_O_20_·*n*H_2_O by substituting Mn with Fe, Co, Ni, Ca, and K are also explored to confirm the unique advantages of transition metal ions.

## 2 Results and Discussion

**Figure 1:**
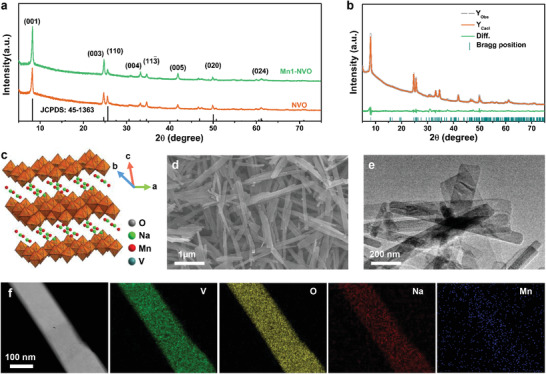
a) XRD patterns of NVO and Mn1‐NVO. b) Rietveld refinement of the XRD pattern of (Na,Mn)V_8_O_20_. c) The crystal structure of Mn1‐NVO. d) SEM image of Mn1‐NVO. e) TEM image of Mn1‐NVO. f) TEM elemental mapping images of Mn1‐NVO nanobelts.

The NVO and Mn‐doped NVO were synthesized via a one‐step hydrothermal method. (Na,Mn)V_8_O_20_·*n*H_2_O with different amounts of Mn content was obtained by setting the molar ratio of MnSO_4_ and V_2_O_5_ to 1:13, 13:13, and 20:13 during the hydrothermal process. The as‐prepared Mn‐doped NaV_8_O_20_·*n*H_2_O was denoted as Mn1‐NVO, Mn2‐NVO, and Mn3‐NVO, respectively. **Figure** **1**a shows the X‐ray diffraction (XRD) patterns of NVO and Mn1‐NVO, in which all the characteristic peaks are well indexed by (Na,Ca)(V,Fe)_8_O_20_·*n*H_2_O with *C*2/*m* space group (JCPDS No. 45‐1363). With the intercalation of a small amount of Mn ion in the NVO, the resulting XRD peaks of Mn1‐NVO were in accord with the structure of NVO, suggesting that the intercalated Mn ions would not produce a new crystal structure. It can be speculated that Mn1‐NVO has two possible structures, which are (Na,Mn)V_8_O_20_·*n*H_2_O and Na(V,Mn)_8_O_20_·*n*H_2_O. The average analytical formula can be written as (Na_0.97_,Mn_0.02_)V_8_O_20_·nH_2_O or Na_0.97_(V_7.98_,Mn_0.02_)O_20_·*n*H_2_O for Mn1‐NVO, as the molar ratio of Na:Mn:V was calculated to be 0.97:0.02:8 based on the electron microprobe analysis (EPMA). The molar ratios of Na:Mn:V for Mn‐doped NVO are shown in Table S1, Supporting Information.

To confirm the position of the Mn ions in NVO, Rietveld refinement of Mn1‐NVO was conducted. The chemical composition of the compound was analyzed by inductively coupled plasma optical emission spectroscopy (ICP‐OES) before progress the Rietveld refinement. The molar ratio of Na:Mn:V was 1.1:0.03:8. Based on the Rietveld refinement results in Figure 1b and Figure S1, Supporting Information, the position of Mn in Mn1‐NVO was confirmed by the reliability factors and the fitting degree of the peak shape in the refinement process. The unit cell parameters for Mn1‐NVO were calculated to be *a* = 11.699(6) Å, *b* = 3.647(8) Å, *c* = 11.119(2) Å, *α* = *γ* = 90°, and *β* = 104.05°. The refinement data in detail are shown in Tables S2 and S3, Supporting Information. After the Mn ion was doped, Na(1) moved away from their average positions and Mn(1) occupied the Na(1) positions, indicating that Mn1‐NVO had the crystal structure of (Na,Mn)V_8_O_20_·*n*H_2_O instead of Na(V,Mn)_8_O_20_·*n*H_2_O (Figure 1c).^[^ ^34^ ^]^ Noted that the occupancy of Na and Mn are not in good coincidence with the ICP and EMPA result, probably because part of Na ions might form interstitial solid solution in Mn1‐NVO and the severe interlayer disorder exist in corvusite.^[^ ^34^ ^]^ The valence of Mn ions in NVO was analyzed via X‐ray photoelectron spectroscopy (XPS), showing a mixture of 83% Mn^3+^ and 17% Mn^2+^ for Mn2‐NVO and Mn3‐NVO (Figure S2 and Table S4, Supporting Information). Noted that the average valence of V in the synthesized NVO was 4.876. While for the Mn‐doped NVO, the average valence of V in (Na_0.97_,Mn_0.02_)V_8_O_20_·*n*H_2_O was 4.871, revealing an increased V^4+^:V^5+^ ratio in this structure. On the contrary, the average valence of V in Na_0.97_(V_7.98_,Mn_0.02_)O_20_·*n*H_2_O was 4.883, suggesting an decreased V^4+^:V^5+^ ratio. From the XPS results (Figure S3 and Table S5, Supporting Information), the V^4+^:V^5+^ ratio of NVO was 0.44:1, and the V^4+^:V^5+^ ratios of Mn1‐NVO, Mn2‐NVO, Mn3‐NVO were 0.47:1, 0.5:1, and 0.53:1, respectively. The increased V^4+^:V^5+^ ratio confirmed that the Mn ion doped NVO had the formula (Na,Mn)V_8_O_20_·*n*H_2_O. With a thermogravimetric‐differential thermal analysis study, the structural water content in Mn1‐NVO was obtained (Figure S4, Supporting Information), and the stoichiometric formula for the prepared Mn1‐NVO was then confirmed as (Na_0.97_,Mn_0.02_)V_8_O_20_·0.32H_2_O.

The scanning electron microscope (SEM) image in Figure 1d reveals a nanobelt morphology for the as‐synthesized Mn1‐NVO, which was a few micrometers in length and several hundred nanometers in width. The transmission electron microscopy (TEM) image in Figure 1e also confirms the microstructure of the Mn1‐NVO material. The homogeneous distributions of V, Na, O, and Mn in the Mn1‐NVO nanobelt were further observed in the TEM elemental mapping images (Figure 1f). The nanobelt morphological features may provide fast kinetics of the Zn^2+^ insertion/extraction in aqueous ZIBs.^[^ ^2^ ^]^

**Figure 2:**
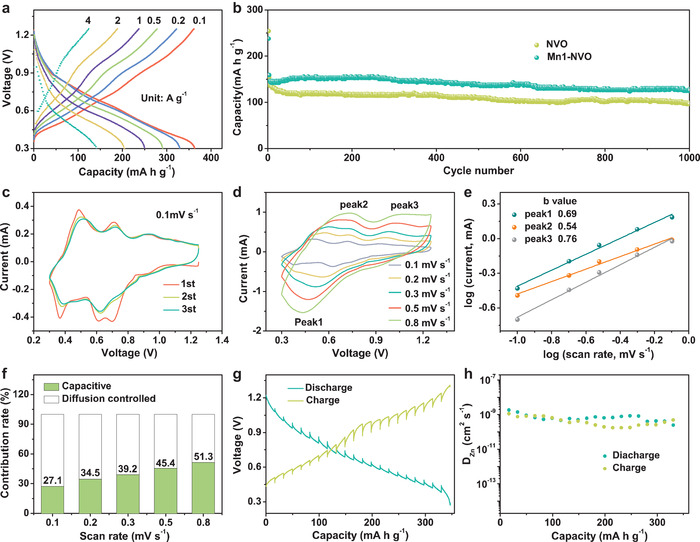
a) Rate performance of Mn1‐NVO electrode. b) Long‐term cycling of Mn1‐NVO electrode at 4 A g^−1^. c) CV curves of Mn1‐NVO at the scan rate of 0.1 mV s^−1^. d) CV curves of Mn1‐NVO electrode at different scan rates. e) log (peak current) versus log (scan rate) plots of each peak shown in d). f) The capacitive contributions at scan rates of 0.1, 0.2, 0.3, 0.5, and 0.8 mV s^−1^. g) Discharge–charge curves of Mn1‐NVO in GITT measurement. h) The diffusivity coefficient of Zn^2+^ in the discharge and charge processes of the Mn1‐NVO in the third cycle.

Using a Zn negative electrode and Ti foil (20 µm) as the positive current collector in 3 m aqueous Zn(CF_3_SO_3_)_2_ electrolyte, the electrochemical performance of the Mn1‐NVO nanobelts as the positive electrode were investigated. The Mn1‐NVO delivers a high capacity of 363 mA h g^−1^ at a current density of 0.1 A g^−1^ (**Figure** **2**a). With the increase in the current density to 1 A g^−1^, the Mn1‐NVO still exhibited a capacity of 251 mA h g^−1^. Mn1‐NVO electrode delivered a capacity of 238 mA h g^−1^ in the first cycle at 4 A g^−1^ (Figure 2b). The specific capacity gradually decreased in the subsequent three cycles and stabilized at 146 mA h g^−1^. It maintained a high capacity of 128 mA h g^−1^ after 1000 cycles with a capacity retention of 88% for Mn1‐NVO, higher than that of 69% for NVO. The related charge and discharge curves for Mn1‐NVO at 4 A g^−1^ are shown in Figure S5, Supporting Information. The initial three cyclic voltammogram (CV) curves of Mn1‐NVO captured at a scan rate of 0.1 mV s^−1^ are shown in Figure 2c. For the Mn1‐NVO electrode, the CV curve at the first cycle was quite different from the second cycle, while its CV curve of the third cycle shows high similarity with the second cycle. During the first cathodic scan, four cathode peaks appeared at 0.94, 0.7, 0.61, and 0.37 V, and the peak at 0.7 V may have been responsible for the irreversible reaction that occurs in the initial cycle. The anodic scans showed four peaks at 0.49, 0.71, 0.92, and 1.1 V. Based on these pairs of reduction/oxidation peaks, a multi‐step reaction mechanism corresponding to the Zn^2+^ insertion/extraction could be inferred, which is quite different from the reported V_2_O_5_ based cathodes that have two pairs of redox peaks.^[^ ^23^ ^]^ Some peaks disappear at a faster scan rate of 0.5 mV s^−1^, leading to one obvious cathode peak at 0.5 V and two anodic peaks at around 0.65 and 1.05 V. Figure 2d displays the CV curves of the Mn1‐NVO batteries at different scan rates with a voltage window from 0.3 to 1.25 V. The peak currents and scan rates have the relationship shown below.^[^ ^34^, ^35^, ^36^, ^37^ ^]^

(1) $$i=av^{b}$$

which can also be written as

(2) $$\log\left(i\right)=b\log\left(v\right)+\log\left(a\right)$$

where the value of *b* in the range of 0.5–1 represents the slope of the log(*i*) versus log(*v*) curve. Ionic diffusion controls the reaction process when the value of *b* is 0.5, and pseudocapacitance dominates the electrochemical process completely when the value of *b* is 1.^[^ ^37^ ^]^ The calculated *b* values of peaks 1, 2, and 3 are 0.69, 0.54, and 0.76, respectively (Figure 2e), meaning the reactions at these peaks are controlled by both ionic diffusion and the capacitive contribution. Furthermore, the capacitive contributions were calculated to be 27.1%, 34.5%, 39.2%, 45.4%, and 51.3% at a scan rate of 0.1, 0.2, 0.3, 0.5, and 0.8 mV s^−1^ as shown in Figure 2f, respectively, revealing that the capacitive contribution increase with the increase in scan rate. It is reported that the increase of capacitive contribution to the whole capacity with the increase in scan rate is controlled by the diffusion of both ion and electron in cathode,^[^ ^14^ ^]^ and the superior rate capability can be attributed to the synergistic contribution from Zn‐ion diffusion and capacitive behavior,^[^ ^14^ ^]^ which may be responsible for the excellent cycling stability at a larger current density for Mn1‐NVO batteries (Figure S6, Supporting Information). In addition, electrochemical impedance spectrum analysis of Mn1‐NVO and NVO batteries after 20 cycles was carried out (Figure S7, Supporting Information). It shows lower charge‐transfer resistance of the Mn1‐NVO than the NVO (Rct 4.8 ohms for Mn1‐NVO versus 14 ohms for NVO) after 20 cycles, which suggests a higher surface electron mobility and improved electrochemical activity for Mn1‐NVO.^[^ ^7^ ^]^

To determine the roles of introducing Mn, the kinetics of the Zn^2+^ diffusion at the Mn1‐NVO and NVO electrodes were studied via the Galvanostatic Intermittent Titration Technique (GITT) measurement, and the corresponding Zn^2+^ diffusion coefficients for the Mn1‐NVO and NVO electrodes in the third cycle are illustrated in Figure 2g,h and Figure S8a,b, Supporting Information. The calculated Zn^2+^ diffusion coefficients in Mn1‐NVO ranged from 1.9 × 10^−9^ to 2.5 × 10^−10^ cm^2^ s^−1^, while the *D*_Zn_^2+^ for NVO ranged from 1.2 × 10^−10^ to 7.0 × 10^−10^ cm^2^ s^−1^ upon discharge. Interestingly, the Zn^2+^‐diffusion coefficient in Mn2‐NVO and Mn3‐NVO (Mn2‐NVO, 10^−9^−10^−10^; Mn3‐NVO, 10^−10^−10^−11^) were only slightly reduced with an increase in Mn content (Figure S8c–f, Supporting Information). The high Zn^2+^ diffusion coefficient may have been responsible for the improved battery performance of Mn1‐NVO. Figure S9, Supporting Information shows Ragone plot of Zn//Mn1‐NVO and Zn//NVO battery. Mn1‐NVO and NVO electrodes achieve an energy density of 249 and 239 Wh kg^−1^ at 100 mA g^−1^, respectively. Even though the Zn//Mn1‐NVO battery cycled at 1 A g^−1^, the energy density still reaches up to 157 Wh kg^−1^ at an outstanding power density of 628 W kg^−1^, which is higher than that of Zn//NVO battery (144 Wh kg^−1^at the power density of 598 W kg^−1^).

**Figure 3:**
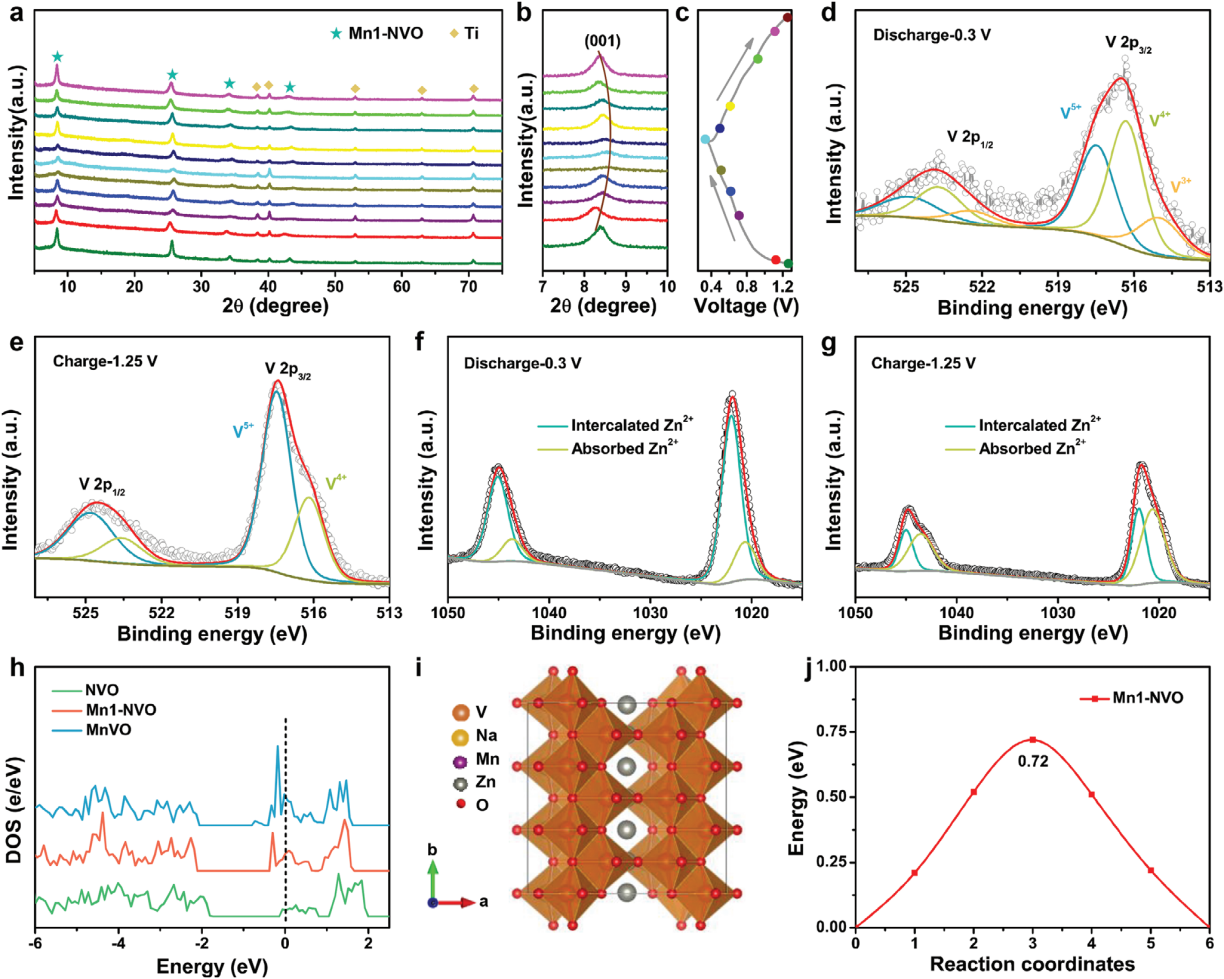
a,b) The ex situ XRD patterns of Mn1‐NVO in the first discharge and charge process. c) The corresponding charge/discharge curves at a current density of 0.05 A g^−1^. d) High resolution XPS spectra of vanadium in fully discharged state. e) High resolution XPS spectra of vanadium in fully charged state. f) High resolution XPS spectra of zinc in fully discharged state. g) High resolution XPS spectra of zinc in fully charged state. h) Density of states of the NVO, Mn1‐NVO, MnVO. i) Possible migration pathways for Zn^2+^ in Mn1‐NVO. j) Energy barriers along Zn^2+^‐migration pathways for Mn1‐NVO.

To investigate the structural evolution and reaction mechanisms of the Mn1‐NVO, ex situ XRD was performed during the first discharge/charge process. As shown in **Figure** **3**a–c, the XRD pattern of the Mn1‐NVO electrode on the Ti foil matches well with the as‐synthesized Mn1‐NVO and Ti current collector in the initial state. When first discharged to 1.1 V at a current density of 0.05 A g^−1^, the diffraction peaks for Mn1‐NVO located at 8.3°, 25.6°, 33.5°, and 43.2° shift to lower angle at 8.2°, 25.3°, 33.8°, and 42.4°, respectively. Particularly, the calculated interlayer spacing of the (001) plane shifts from 10.62 Å to 10.70 Å during the initial Zn^2+^ insertion process, which is ascribed to the insertion of a certain amount of Zn^2+^ into the interlayer of Mn1‐NVO, expanding the interlayer spacing. Notably, the interlayer spacing of the (001) plane shifts to 10.46 Å and 10.31 Å upon the following discharge to 0.6 and 0.3 V, indicating that lattice contraction occurs. At the subsequent charging from 0.3 to 0.5 V, the diffraction peaks located at 8.6°, 25.8°, 34.6°, and 43.8° shift to a lower angle at 8.5°, 25.9°, 34.5°, and 43.6°, respectively, suggesting an increase in interlayer space corresponding to the deintercalation of Zn^2+^. When first charged to 1.25 V, the interlayer spacing of the (001) plane increased from 10.31 Å to 10.54 Å. The fully charged electrode at 1.25 V after 400 cycles showed the same XRD pattern as the electrode cycled at the tenth cycle (Figure S10, Supporting Information), suggesting that a reversible phase transition occurred during long‐term cycling.

To confirm whether there is new phases formation upon discharge/charge, Raman spectra were provided to further show structural information on the Mn1‐NVO electrode at the fully discharged/charged states. As shown in Figure S11, Supporting Information, the strong Raman shift at around 846 cm^−1^ is ascribed to V‐O‐Zn vibrations in Mn1‐NVO upon discharge, and the Raman shifts at 253, 310 and 371 cm^−1^ are assigned to the Zn‐OH vibrations, implying the presence of Zn(OH)_2_ or Zn*_x_*(OTf)*_y_*(OH)_2_ *_x_*_−_ *_y_*·*n*H_2_O upon fully discharge.^[^ ^43^ ^]^ The OH^−^ in the compound is generated from the decomposition of water, thus accompany with the formation of H^+^, and H^+^ may intercalate in the Mn1‐NVO electrode to maintain a neutral charge state.^[^ ^44^, ^45^ ^]^ Therefore, the reaction mechanism can be inferred that the H^+^ is co‐intercalated with Zn^2+^ into Mn1‐NVO upon discharge from the presence of the above precipitates. However, the low crystallinity and small quantity of the precipitates may be the reason why XRD cannot detect the phase. As shown in the enlarged XRD patterns in Figure S12, Supporting Information, there are no extra signals besides peaks of Mn1‐NVO, suggesting that the main structure is maintained during the charging/discharging process. The lowering of the relative intensity and the broadening of the XRD peaks during the discharge was mainly due to the formation of Zn(OH)_2_ and Zn*_x_*(OTf)_y_(OH)_2_ *_x_*_−_ *_y_*·*n*H_2_O precipitates and the insertion of Zn^2+^ into the Mn1‐NVO. In addition, the peak associated with V—O—Zn bond and the characteristic Raman peaks of precipitates disappear at the fully charged state, and the spectra is similar with the pristine electrode, suggesting there is no Zn complex formed upon charge. Upon fully charged state, the recovery of the XRD peak was due to the extraction of Zn^2+^ and H^+^ from the Mn1‐NVO cathode, which is in accord with the previous studies.^[^ ^44^, ^45^ ^]^ Noted that the energy storage mechanism of Mn1‐NVO involves mainly Zn^2+^ (de)intercalation with minor H^+^ (de)intercalation, which is in accord with the previous report.^[^ ^46^ ^]^ For Zn anodes, the Raman shifts at the fully charged/discharged state are similar with the pristine Zn anodes, and the peak associated with O‐Zn bond is stable during the cycling, suggesting there is no Zn complex formed upon charge.

The storage of Zn^2+^ in the Mn1‐NVO electrode was further demonstrated by ex situ high‐resolution XPS analysis. Characteristic peaks at 517.5 and 516.2 eV in Figure S3b, Supporting Information showed the V^5+^ and V^4+^ for Mn1‐NVO.^[^ ^23^ ^]^ As shown in Figure 3d, the 2p peaks of V^5+^ become weak, while the peaks for V^4+^ and V^3+^ increase during the intercalation of Zn^2+^, indicating the electrochemical reduction of V cations in the V_8_O_20_ framework during the first discharge. Upon charging in Figure 3e, the intensity of V^5+^ increases as V cations are oxidized to +5, and the molar ratio of V^5+^:V^4+^ recovers to that of the pristine V spectrum (0.68:0.32; Table S6, Supporting Information). Meanwhile, the Zn 2p peaks emerge at the discharge state of 0.3 V (Figure 3f and Table S7, Supporting Information). The Zn 2p_3/2_ appears at 1022 and 1020.7 eV, corresponding to the intercalated Zn and absorbed Zn during discharge.^[^ ^29^ ^]^ As mentioned above, there is Zn complex (such as Zn(OH)_2_ or Zn*_x_*(OTf)*_y_*(OH)_2_ *_x_*_−_ *_y_*·*n*H_2_O) formed on the cathode upon the fully discharge state. The Zn 2p signals located at 1022 eV at fully discharged state was caused by the intercalated Zn and the zinc hydroxide triflate precipitation.^[^ ^29^, ^45^ ^]^ Upon charging, the intensity of the Zn peaks remarkably decreases (Figure 3g), but the intercalated Zn (Zn 2p_3/2_, 1022 eV) and absorbed Zn features remain (Zn 2p_3/2_, 1020.7 eV; Table S7, Supporting Information). As a result, the NVO crystal structure consists of a small amount of lattice‐trapped Zn after the first cycle, which might be the reason for the rapidly declining capacity in the second cycle. Mn ions were detected by XPS in the fully discharged and charged states both in the first cycle and the 400th cycle (Figure S13, Supporting Information), meaning Mn ions keep stable in the crystal structure during the cycling. The Mn ions existing in the NVO crystal structure can act as pillar and stabilize the structure during the long‐term cycling. Furthermore, the Mn ions can change the electronic structure by strengthening the chemical bonds, thus causing more stable cycling performance than that in NVO. To confirm the electronic structure change of Mn1‐NVO, conductivity of the samples was carried out. The Mn1‐NVO exhibits higher electrical conductivity than NVO (Figure S14, Supporting Information). To show the role of Mn ion and Na ion in Mn1‐NVO more clearly, the theoretical calculations of density of states and Zn^2+^ diffusion pathways for the NaV_8_O_20_, (Na,Mn)V_8_O_20_, MnV_8_O_20_ (MnVO) are provided in Figure 3h–j and Figures S15 and S16, Supporting Information. Density functional theory (DFT) calculation results in Figure 3h show that there is an increased electron density near the Fermi level for Mn1‐NVO than that of NVO, suggesting Mn ions lead a higher electrical conductivity than Na ions. Undoubtedly, a higher electrical conductivity can boost the electron transfer during Zn^2+^ (de)intercalation, thus contributes to the excellent performance of Mn‐doped NVO.^[^ ^29^ ^]^ Possible migration pathways for Zn^2+^ in Mn1‐NVO is shown in Figure 3i, where Zn^2+^ mainly diffuses along *b* axis. Energy barriers along Zn^2+^‐migration pathways for NVO and Mn1‐NVO is lower than that of MnVO (Figure 3j), suggesting Na ions cause a faster Zn^2+^‐migration than Mn ions. The outstanding performance of Mn1‐NVO can be attributed to the synergistic contribution from Na ions and Mn ions.

**Figure 4:**
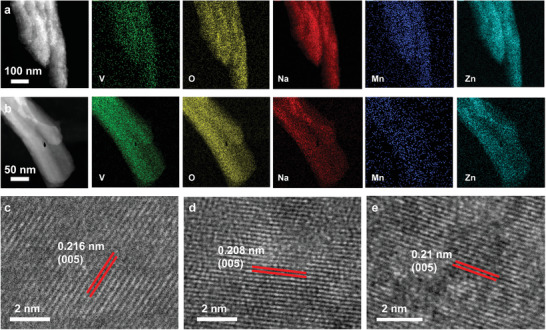
a) TEM‐EDX element mapping images in fully discharged state of the Mn1‐NVO electrode. b) TEM‐EDX element mapping images in fully charged state of the Mn1‐NVO electrode. c) HRTEM image of pristine Mn1‐NVO. d) HRTEM image of the Mn1‐NVO electrode at fully discharged state. e) HRTEM image of the Mn1‐NVO electrode at fully charged state.

To gain better insight into the electrochemical process during charging/discharging, the morphologies after the first discharge and charge were observed by TEM. The nanobelts was maintained during the cycling, and the element distributions of V, O, Na, Mn, and Zn was collected by EDS mapping with TEM (**Figure** **4**a,b). The element distribution of Zn illustrates the insertion of Zn^2+^ into the Mn1‐NVO lattice. A weaker signal from Zn was detected at the charged state compared with the discharged state, meaning that the lattice trapped Zn, and the absorbed Zn ions exited after the Zn^2+^ extraction, which agrees well with the previous XPS analysis.^[^ ^2^, ^29^, ^38^, ^45^ ^]^ The high‐resolution‐TEM (HRTEM) images in Figure 4c–e further confirmed the contraction of lattice spacing during the first discharge. The TEM image in Figure 4c confirmed the crystallinity of the Mn1‐NVO material. A lattice fringe with spacing of 0.216 nm can be observed in the HRTEM image, which corresponds to the d‐spacing of the (005) planes of the Mn1‐NVO nanobelt. The contraction (≈3.7%) of the Mn1‐NVO lattice was observed upon the first discharge, as the spacing of the (005) planes decreased from 0.216 to 0.208 nm. This is likely attributed to the increased screening of the interlayer electrostatic repulsion as demonstrated in previous reports.^[^ ^2^, ^29^ ^]^ Upon charging, the lattice spacing increased to 0.21 nm with Zn^2+^ extraction, which was in accord with the ex situ XRD results.

**Figure 5:**
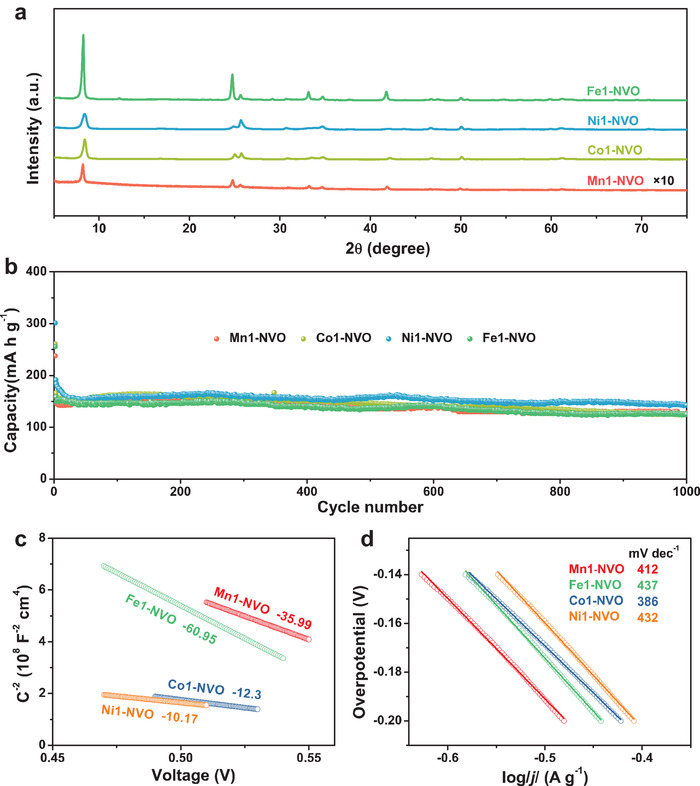
a) XRD patterns of as synthesized Fe1‐NVO, Co1‐NVO, and Ni1‐NVO. b) Cycle performance at a current rate of 4 A g^−1^. c) Mott–Schottky plots of Mn1‐NVO, Fe1‐NVO, Co1‐NVO, and Ni1‐NVO. d) Tafel curves of Mn1‐NVO, Fe1‐NVO, Co1‐NVO, and Ni1‐NVO at a sweep rate of 1 mV s^−1^.

To explore the reasons why the Mn ions stabilized the NVO structure, other metal sulfates, such as FeSO_4_, CoSO_4_, NiSO_4_, CaSO_4_ and K_2_SO_4_, were used as raw materials to replace MnSO_4_, producing (Na,Fe)V_8_O_20_, (Na,Co)V_8_O_20_, (Na,Ni)V_8_O_20_, (Na,Ca)V_8_O_20_, and (Na,K)V_8_O_20_ (denoted as Fe1‐NVO, Co1‐NVO, Ni1‐NVO, Ca1‐NVO, and K1‐NVO), respectively. The XRD results in **Figure** **5**a and Figure S17, Supporting Information indicated that these synthesized cathodes maintained the NVO structure. Similar to Mn1‐NVO, the cycle life of Fe1‐NVO, Co1‐NVO, Ni1‐NVO, Ca1‐NVO, and K1‐NVO at 4 A g^−1^ was investigated. An initial decrease in capacity was observed for these cathodes, which might be attributable to the slight surface dissolution of materials and the irreversible reactions during the first few cycles. Taking Fe1‐NVO as an example (Figure 5b), a discharge capacity of 149 mA h g^−1^ was delivered after the first three cycles for Fe‐NVO, and 84% of the capacity was achieved after 1000 cycles, displaying a much better cycling performance than NVO. Meanwhile, the cycling performance of Ca1‐NVO and K1‐NVO were poorer than Fe1‐NVO and Mn1‐NVO. For example, The Ca1‐NVO and K1‐NVO electrodes exhibited an excellent cycling stability during the first few cycles but suffered from lower capacity (Figure S18a,b, Supporting Information). To further understand the enhanced cycle stability of NVO via transition metal insertion, Mott–Schottky plots and Tafel curves are shown in Figure 5c,d. The negatively sloped Mott–Schottky plots of NVO and Mn1‐NVO imply a p‐type semiconductor character.^[^ ^39^, ^40^ ^]^ Furthermore, there is a decreasing trend in the absolute value of the slope for transition metal‐doped NVO compared with pristine NVO, Ca1‐NVO, and K1‐NVO (Figure 5c and Figure S18c, Supporting Information), indicating that the M1‐NVO (M = Mn, Fe, Co, Ni) electrodes offer higher charge carrier density than the NVO, Ca1‐NVO, and K1‐NVO. The slopes of the Tafel curves of Ca1‐NVO, K1‐NVO, Mn1‐NVO, Fe1‐NVO, Co1‐NVO, and Ni1‐NVO were 491, 475, 412, 437, 386, and 432 mV dec^−1^ (Figure 5d and Figure S18d, Supporting Information), respectively. Obviously, decreased slopes of the Tafel curves for M1‐NVO (M = Mn, Fe, Co, and Ni) were observed, which might be attributable to the catalytic effect of transition metal cations originating from the partially unfilled 3d orbitals that could transfer electrons and accelerate the redox reaction during the Zn^2+^ storage reaction.^[^ ^23^, ^41^, ^42^ ^]^ Furthermore, other cathode materials that were derived from (Na,Mn)V_8_O_20_·*n*H_2_O by substituting Na with Li, K, and Zn (denoted as Mn1‐LVO, Mn1‐KVO, Mn1‐ZVO) were also explored to confirm the catalytic effect of Mn ion. As shown in Figure S19a,b, Supporting Information, the crystal structures of these derivates were similar to that of Mn1‐NVO, and these cathodes exhibited more outstanding cycling performances compared with that of NVO.

**Figure 6:**
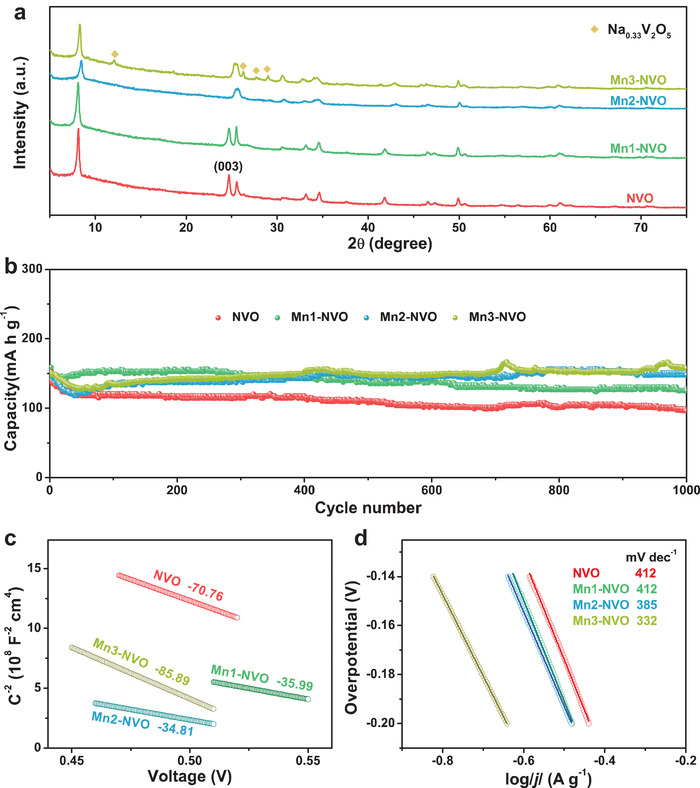
a) XRD patterns of as synthesized NVO, Mn1‐NVO, Mn2‐NVO, and Mn3‐NVO. b) Long‐term cycling performance of NVO, Mn1‐NVO, Mn2‐NVO, and Mn3‐NVO at a current rate of 4 A g^−1^. c) Mott–Schottky plots of NVO, Mn1‐NVO, Mn2‐NVO, and Mn3‐NVO. d) Tafel curves of NVO, Mn1‐NVO, Mn2‐NVO, and Mn3‐NVO at a sweep rate of 1 mV s^−1^.

To further improve the cycling performance of the Zn//Mn1‐NVO battery, the content of Mn in NVO was carefully investigated. With an increase in Mn content in NVO (e.g., Mn2‐NVO, Mn3‐NVO), we found that the (003) plane disappear (**Figure** **6**a). Another phase of Na_0.33_V_2_O_5_ appeared in the Mn3‐NVO. Interestingly, Mn2‐NVO as well as the mixture of (Na,Mn)V_8_O_20_ and Na_0.33_V_2_O_5_ as cathodes exhibited more outstanding cycling performances than Mn1‐NVO (Figure 6b). Obviously, the higher charge carrier density and decreased slope of the Tafel curve for Mn2‐NVO were observed than that of Mn1‐NVO (Figure 6c,d). From this point of view, we deduced that a larger amount of Mn ions in the NVO could stabilize the crystal structure more effectively, thus causing the Mn2‐NVO cathode to exhibit outstanding electrochemical performance with a capacity of 150 mA h g^−1^ at 4 A g^−1^ and a capacity retention of 99% over 1000 cycles. The cycling performances of the developed NVO‐based electrodes compared with previously reported cathodes are listed in Table S8, Supporting Information and show excellent capacity retention during long‐term cycling. Note that the intercalation of a large amount of Mn ions into the NVO during the hydrothermal process has a reverse effect on the (001) lattice spacing, that is, the lattice spacing is 10.46 Å in Mn2‐NVO and 10.62 Å in Mn3‐NVO, respectively. Thus, the combined effects of proper lattice spacing, as well as the stable crystal structure are key factors that determine electrochemical performance. In addition, the capacitive contribution of Mn2‐NVO was calculated to be 37.2%, 45.5%, 50.6%, 56.9%, and 62.6% at the scan rate of 0.1, 0.2, 0.3, 0.5, and 0.8 mV s^−1^, respectively, which is higher than that of Mn1‐NVO or NVO (Figure S20, Supporting Information). The capacitive contribution increased with the increase in Mn content in NVO, and this was also responsible for the excellent cycling performance of the Mn2‐NVO and Mn3‐NVO batteries.

Since the dissolution of NaV_3_O_8_ in the ZnSO_4_ electrolyte and the formation of Zn dendrites caused rapid capacity fading,^[^ ^27^ ^]^ we carefully studied the dissolution of NVO and Mn‐doped NVO electrodes. To confirm the dissolution of NVO and Mn‐doped NVO, the NVO and Mn1‐NVO electrodes were dipped in the 1 m ZnSO_4_ electrolyte for 12 h. ICP‐OES results confirmed the dissolution of NVO and Mn1‐NVO (Table S9, Supporting Information). Furthermore, the dissolution of Mn1‐NVO could be suppressed via the addition of 2 m Na_2_SO_4_ to the 1 m ZnSO_4_, and therefore the capacity retention of Zn//Mn1‐NVO is superior to that of the case without Na_2_SO_4_ (Figure S21a, Supporting Information), which was in accord with the previous report.^[^ ^27^ ^]^ Noted that a high level of Na_2_SO_4_ additive in the electrolyte decreases the energy density of the batteries when they are used in practical applications. As shown in Figure S21, Supporting Information, increasing the concentration of Na_2_SO_4_ can suppress the dissolution of Mn1‐NVO and show high capacity retention, but the capacity of Mn1‐NVO was decreased. The dissolution of Mn1‐NVO in 1, 2, and 3 m ZnSO_4_ electrolytes was also carried out. As shown in Figure S22a, Supporting Information, a high concentration of electrolytes can delay the dissolution process. More importantly, it was noted that the dissolution of Mn‐NVO in ZnSO_4_ electrolytes was suppressed with the increase in Mn content in NVO, which is likely attributed to the increased structural stability. The increasing Mn in NVO causes the electrolyte colors to become faint, as shown in Figure S22b, Supporting Information. Interestingly, the dissolution of Mn1‐LVO, Mn1‐KVO, and Mn1‐ZVO in 1 m aqueous ZnSO_4_ was slower than that of Mn1‐NVO (Figure S22c, Supporting Information), suggesting that Na ions play a significant role in the dissolution process. Furthermore, different kinds of doping transition metals also affect the dissolution process of NVO electrodes (Figure S22d, Supporting Information), as Fe1‐NVO and Ni1‐NVO have a slower dissolution rate than Mn1‐NVO or Co1‐NVO. The dissolution of Mn1‐NVO in 3 m aqueous Zn(CF_3_SO_3_)_2_ was also carried out to show the advantage of Zn(CF_3_SO_3_)_2_ electrolyte for Mn1‐NVO (Figure S22a, Supporting Information). As shown in Figure S23, Supporting Information, a large number of vertical and harsh Zn dendrites formed on the surface of Zn negative electrode in the ZnSO_4_ electrolyte after 500 cycles at 4 A g^−1^, while no obvious Zn dendrites in 3M Zn(CF_3_SO_3_)_2_ is observed. Therefore, compared with ZnSO_4_ electrolyte, 3M Zn(CF_3_SO_3_)_2_ can not only suppress dissolution of Mn1‐NVO, but also suppress the formation of vertical and harsh Zn dendrites during the cycling.

## 3 Conclusion

In summary, we reported a series of transition metal‐doped NVO, in which transition metal ions were intercalated in the interlayers of V_8_O_20_ framework. The Mn1‐NVO used as a cathode delivered a high capacity retention of 88% after 1000 cycles at a high current density of 4 A g^−1^, which was much better than NVO, Ca1‐NVO, or K1‐NVO. With an increase in Mn content in NVO, (Na_0.33_,Mn_0.65_)V_8_O_20_ ·*n*H_2_O can deliver a reversible capacity of 150 mA h g^−1^ and a capacity retention of 99% after 1000 cycles. Importantly, the dissolution of Mn‐doped NVO was suppressed with an increase in the Mn ion content, providing another way to stabilize the cathodes in the electrolyte. When combined with improved ion diffusion kinetics, charge carrier concentration, and catalysis of transition metal ions, the electrochemical performance of NVO‐based cathodes significantly improved. This strategy may pave the way for the potential application of doping in cathode materials and expand the choice of cathodes for energy storage devices.

## 4 Experimental Section

4.1

4.1.1

##### 4.1.1.1 Synthesis

The Mn1‐NVO nanobelts were prepared by one step hydrothermal method. Briefly, 4.73 g of V_2_O_5_, 1.99 g of Na_2_SO_4_, and 0.676 g of MnSO_4_·H_2_O were dispersed in 50 mL of DI H_2_O, then CH_3_OOH was used to adjust pH to 3. After that, the solution was transferred into a 100 mL Teflon‐lined stainless‐steel autoclave. The sealed autoclave was heated to 180 °C for 72 h. Finally, the Mn1‐NVO sample was collected by centrifugation and washed with water and ethanol for three times. The sample was dried at 60 °C in an electric oven for 6 h, then further dried at 60 °C in a vacuum oven for 12 h. NVO was synthesized with the same procedure and processing conditions without MnSO_4_ source.

##### 4.1.1.2 Material Characterization

XRD was performed using a SmartLab‐3KW with Cu K*α* radiation to determine the phase of samples. The morphologies were characterized by SEM (S‐4800) and TEM (JEM‐2100F). XPS (ESCALAB 250) with Al K*α* X‐ray radiation was carried out to measure the oxidation states of the elements. The structural water was detected by the thermogravimetric analysis (LABSYS EVO) under nitrogen atmosphere with a rate of 10 °C min^−1^ from room temperature to 800 °C and holding at 800 °C for 30 min and then cooling naturally to room temperature. ICP (iCAP Q) performed to analyze the elementals ratio.

##### 4.1.1.3 Electrochemical Measurement

The electrodes were assembled into coin cells (CR2032) to investigate the electrochemical performance. Typically, the active material, acetylene black and polyvinylidene fluoride with a mass ratio of 7:1.5:1.5 were fully mixed in N‐methyl‐2‐pyrrolidone solvent for the electrode preparation. Then the slurry was pasted on a Ti foil and dried in a vacuum oven at 60 °C for 12 h. The active material mass loading was around 2 mg cm^−2^. Zinc foil as the anode and glass fiber membrane was used as the separator. LAND battery testing system (CT2001A) was used to evaluate the electrochemical performance of the assembled cells with a voltage range from 0.3 to 1.25 V versus Zn/Zn^2+^. CV, Tafel plot, and Mott–Schottky plots were collected by using a CHI 600E electrochemical workstation.

## 6 Conflict of Interest

The authors declare no conflict of interest.

## Supporting information

Supporting Information
