# Supplementary material for: Tunable Layered (Na,Mn)V8O20·nH2O Cathode Material for High‐Performance Aqueous Zinc Ion Batteries
Source: Adv Sci (Weinh). 2020 May 28;7(13):2000083. doi: 10.1002/advs.202000083 (PMC7341090; doi:10.1002/advs.202000083)
Supplement: Supplementary file 1 — Supporting Information [file ADVS-7-2000083-s001.pdf]

((Supporting Information can be included here using this template))

Copyright WILEY-VCH Verlag GmbH & Co. KGaA, 69469 Weinheim, Germany,

2020.

Supporting Information

Tunable Layered (Na,Mn)$V_8O_{20}\cdot nH_2O$ Cathode Material for High-Performance Aqueous Zinc Ion Batteries

Min Du, Chaofeng Liu, Feng Zhang, Wentao Dong, Xiaofei Zhang, Yuanhua Sang, Jian-Jun Wang, Yu-Guo Guo, Hong Liu*, Shuhua Wang**

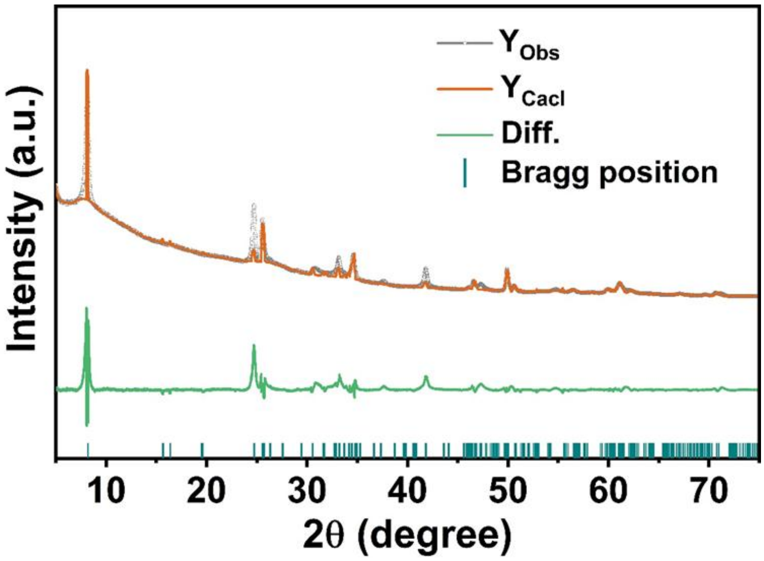

Figure S1. Rietveld refinement of the XRD pattern of $Na(V,Mn)_8O_{20}$.

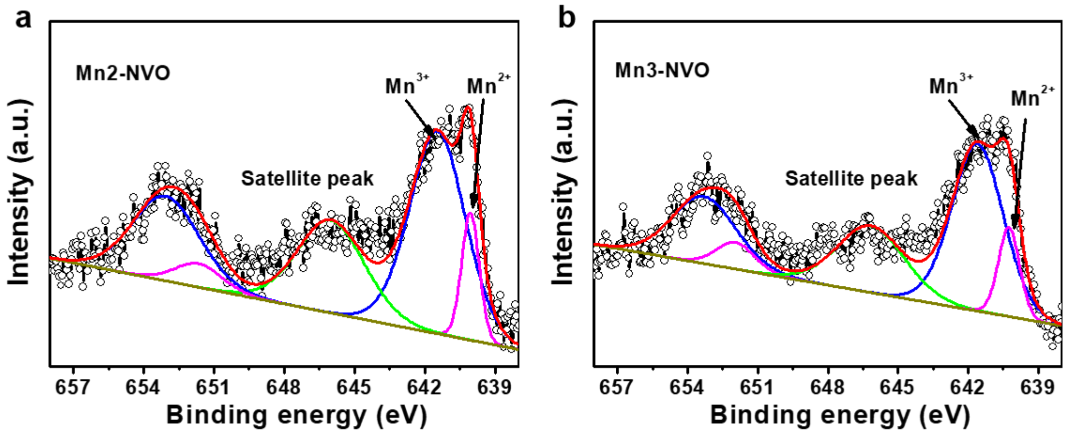

Figure S2. a) High resolution XPS spectra of manganese in pristine Mn2-NVO. b) High resolution XPS spectra of manganese in pristine Mn3-NVO.

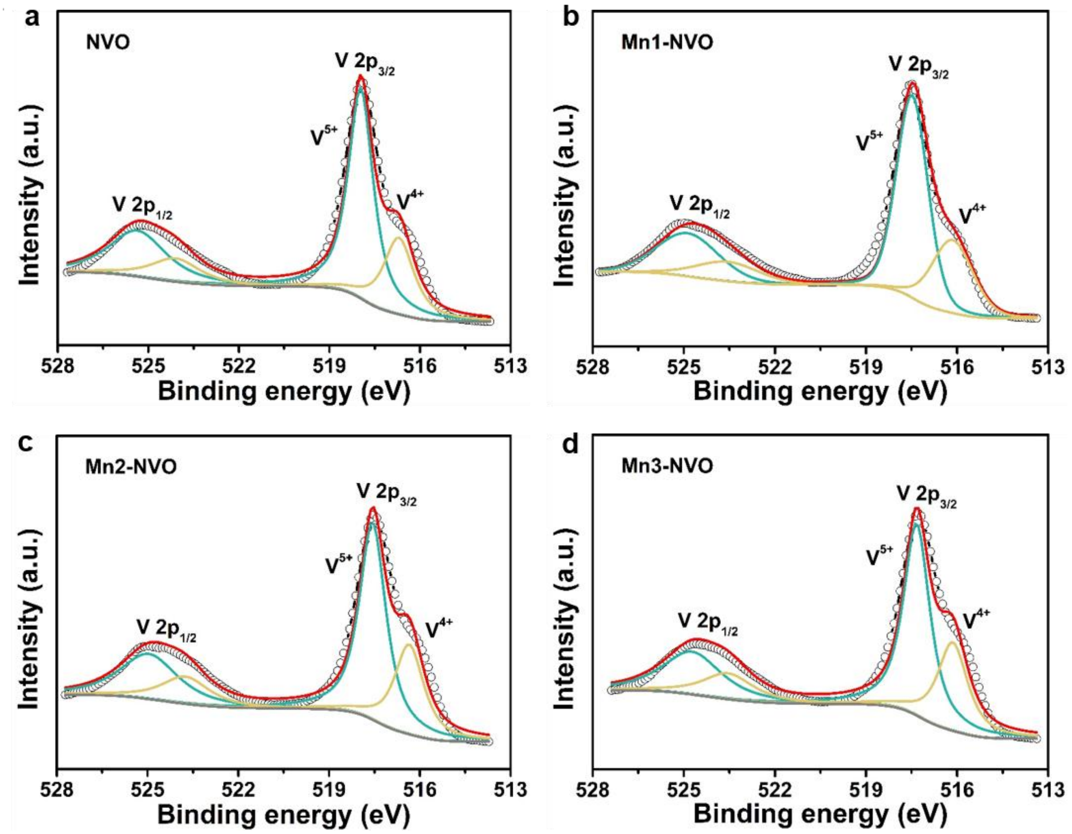

Figure S3. a) High resolution XPS spectra of vanadium in pristine NVO. b) High resolution XPS spectra of vanadium in pristine Mn1-NVO. c) High resolution XPS spectra of vanadium in pristine Mn2-NVO. d) High resolution XPS spectra of vanadium in pristine Mn3-NVO.

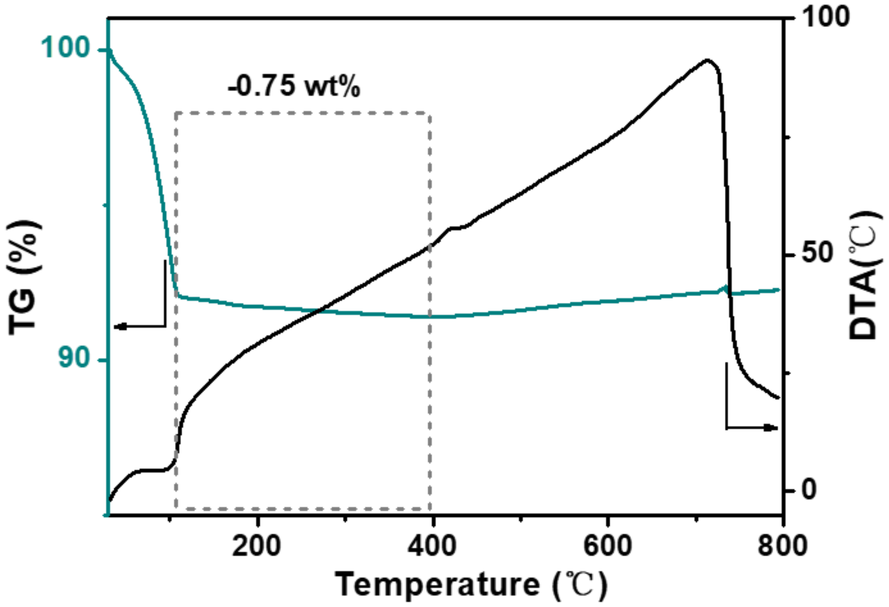

Figure S4. TG-DTA result for Mn1-NVO electrode.

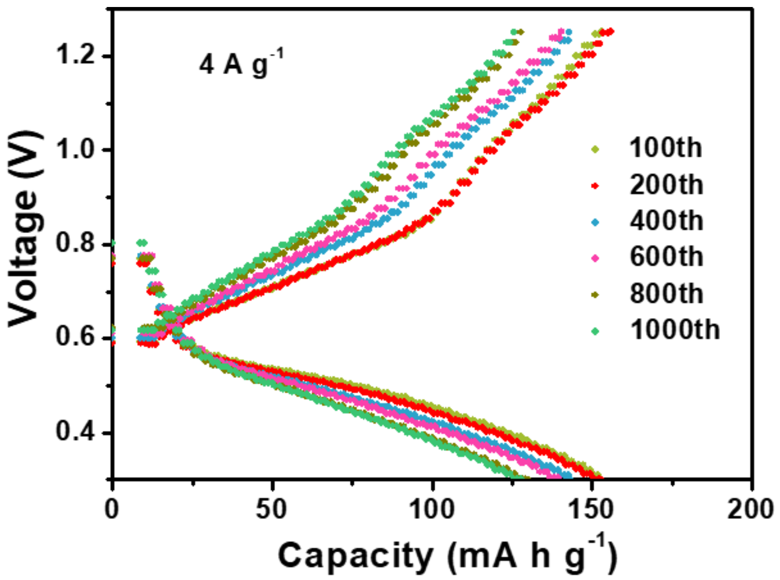

Figure S5. Voltage profiles of Mn1-NVO at 4 A g^{-1}.

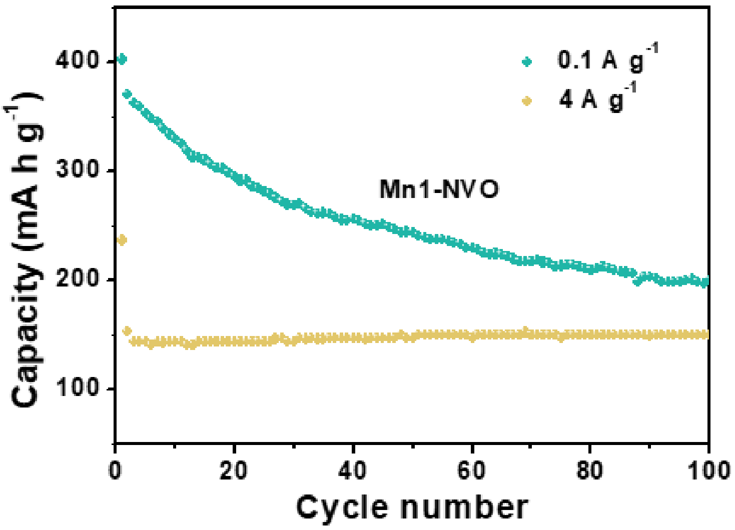

Figure S6. Long-term stability of Zn/Mn1-NVO batteries at different current densities of 0.1 A g^{-1} and 4 A g^{-1}.

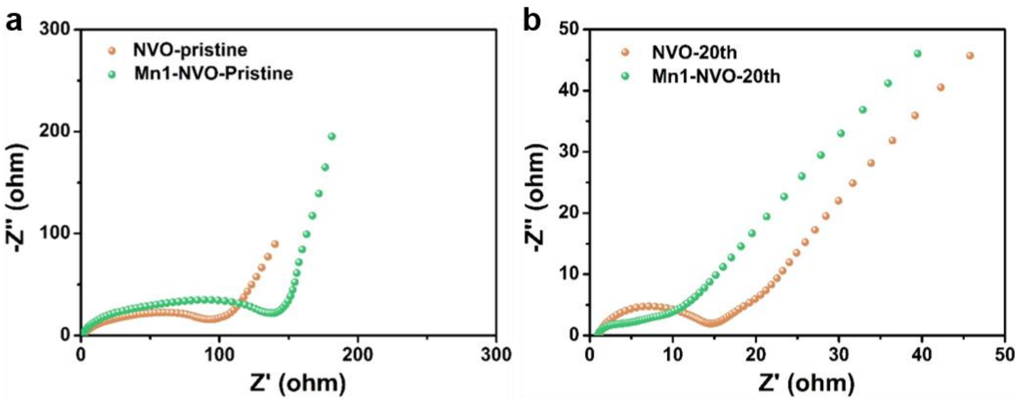

Figure S7. a) EIS spectrum of Mn1-NVO and NVO batteries after cycles of 0 (original). b) EIS spectrum of Mn1-NVO and NVO batteries after 20 cycles. The charge-transfer resistances of NVO and Mn1-NVO batteries are 92 and 137 ohms. It shows lower charge-transfer resistance of the Mn-NVO than the NVO (Rct 4.8 ohms for Mn-NVO vs 14 ohms for NVO) after 20 cycles.

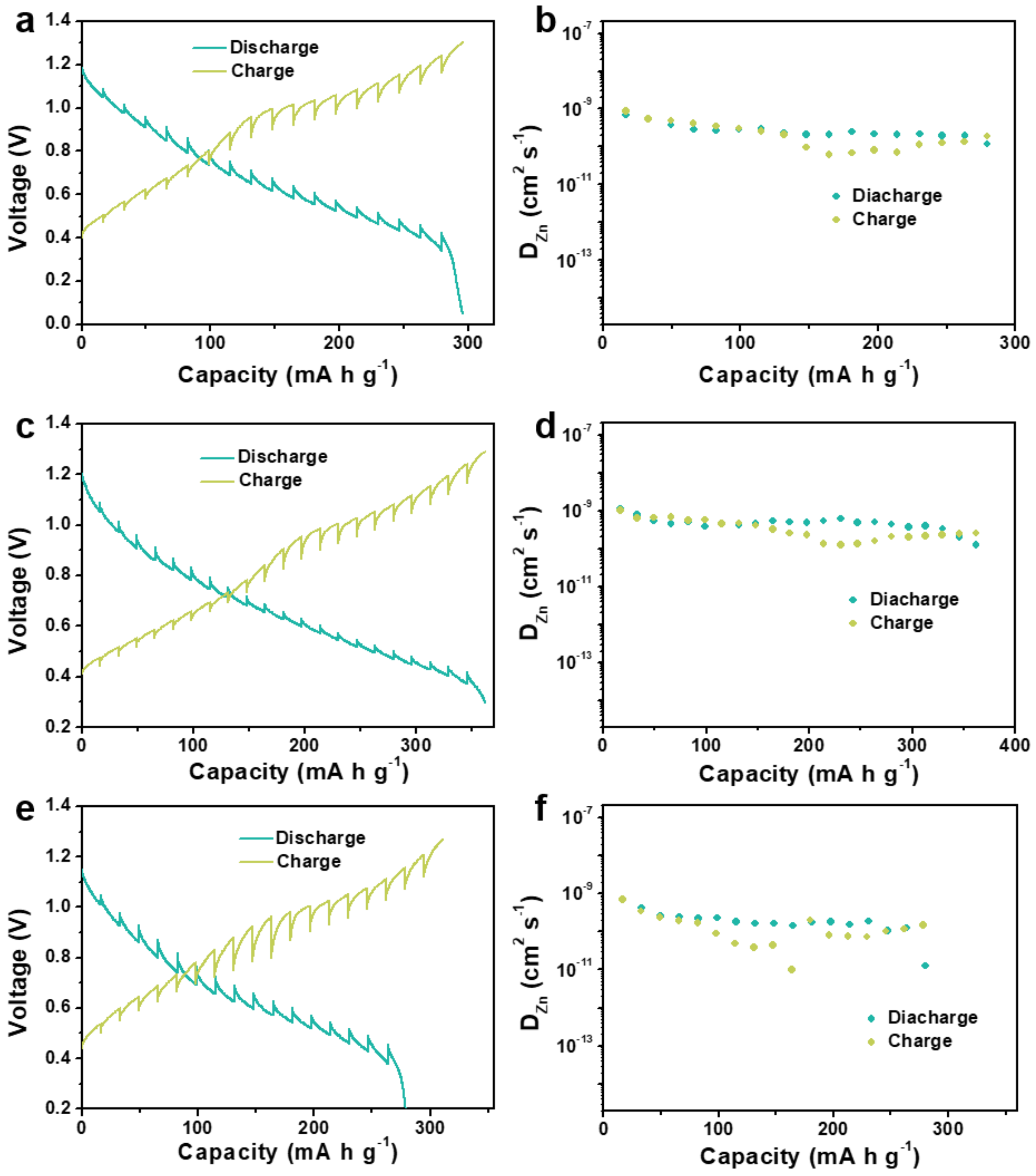

Figure S8. a) Discharge-charge curves of NVO in GITT measurement. b) The diffusivity coefficient of Zn^{2+} in the discharge and charge processes of the NVO at the third cycle. c) Discharge-charge curves of Mn2-NVO in GITT measurement. d) The diffusivity coefficient of Zn^{2+} in the discharge and charge processes of the Mn2-NVO at the third cycle. e) Discharge-charge curves of Mn3-NVO in GITT measurement. f) The diffusivity coefficient of Zn^{2+} in the discharge and charge processes of the Mn3-NVO at the third cycle.

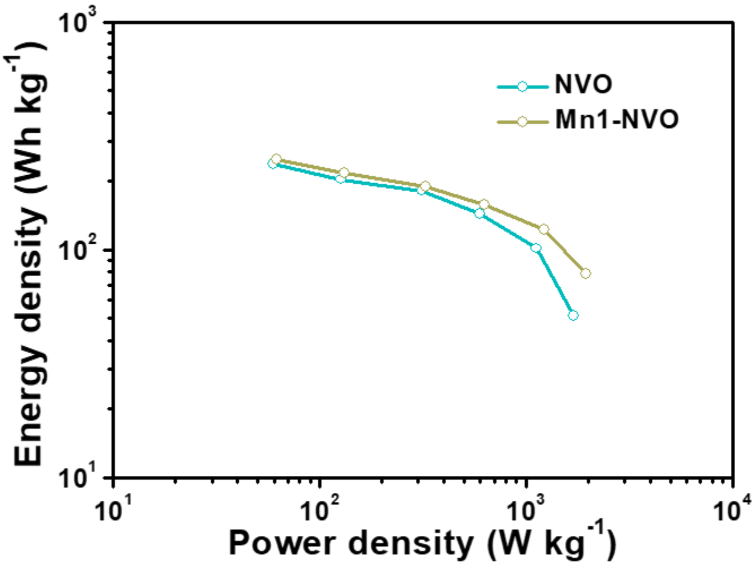

Figure S9. Ragone plots of Zn//Mn1-NVO and Zn//NVO battery. NVO and Mn1-NVO electrodes achieve an energy density of 239 and 249 Wh kg^{-1} at 100 mA g^{-1}, respectively. When the Zn//Mn1-NVO battery cycled at 1 A g^{-1}, the energy density is as high as 157 Wh kg^{-1} at an outstanding power densities of 628 W kg^{-1}, which is higher than that of Zn//NVO battery (144 Wh kg^{-1}at the power density of 598 W kg^{-1}).

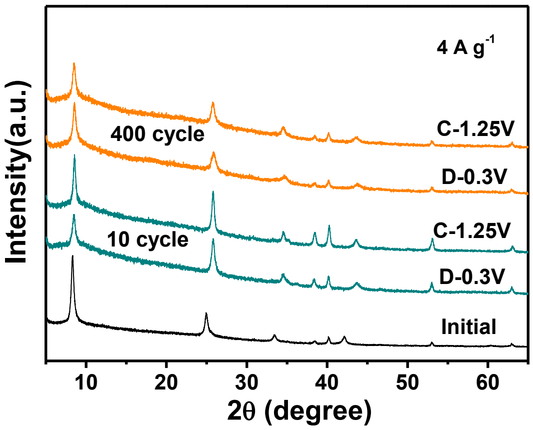

Figure S10. Ex-situ XRD patterns of Mn1-NVO at the 10th cycle and 400th cycle.

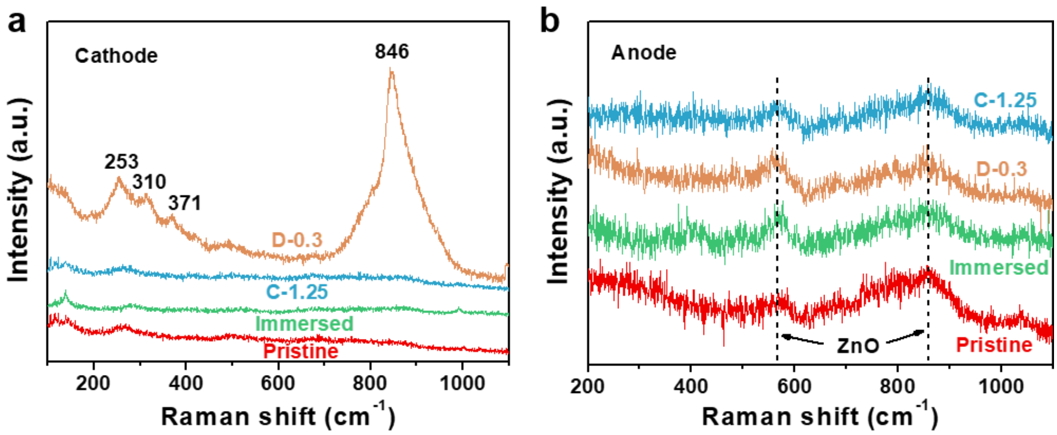

Figure S11. Raman spectra of a) Mn1-NVO cathode, b) Zn anode at pristine, fully discharged/charged states and after immersed into 3M $Zn(CF_3SO_3)_2$, respectively.

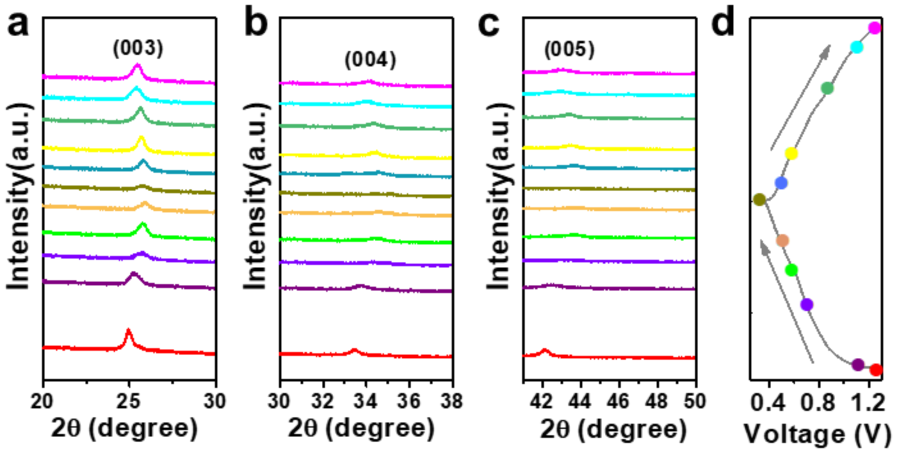

Figure S12. a-c) The enlarged XRD patterns of Mn1-NVO during the first cycle at 0.05 A g^{-1} at different discharge and charge potentials. d) The corresponding charge/discharge curves for a-c.

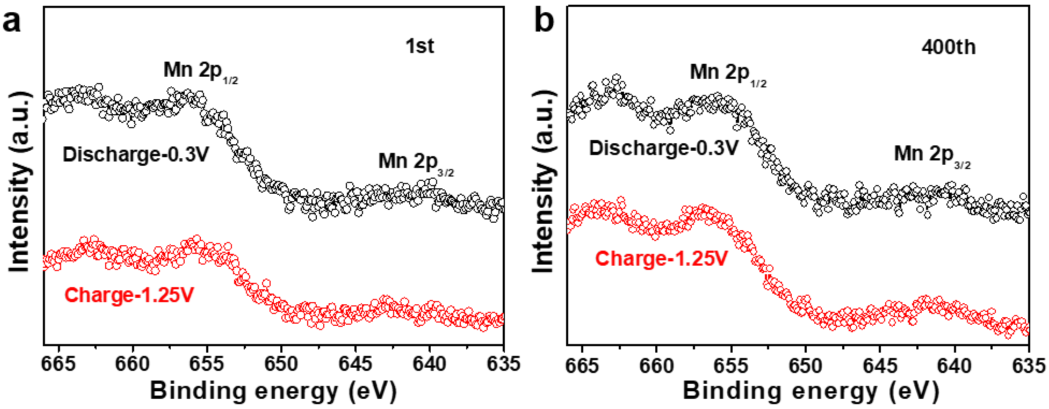

Figure S13. a) High resolution XPS spectra of manganese in Mn1-NVO at the first fully discharged and charged state. b) High resolution XPS spectra of manganese in Mn1-NVO at the 400th fully discharged and charged state.

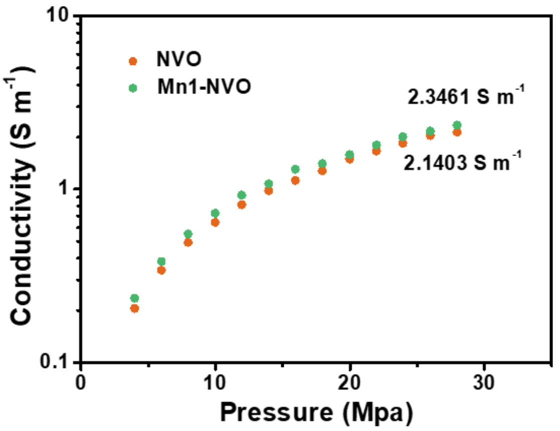

Figure S14. The electrical conductivities of NVO and Mn1-NVO.

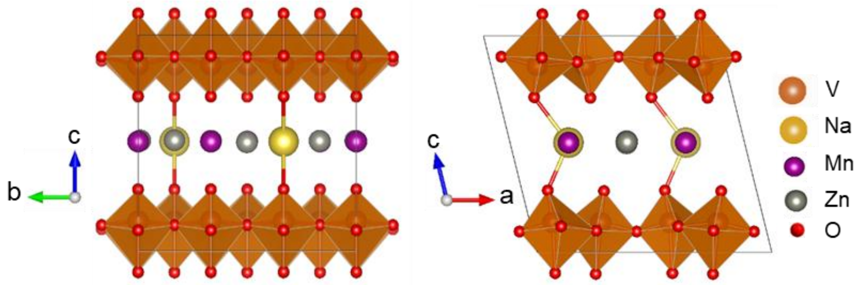

Figure S15. Possible migration pathways for Zn^{2+} in Mn1-NVO. In this work, first-principles calculations are performed based on density-functional theory (DFT) by using Vienna *Ab initio* Simulation Package (VASP) with the projector augmented wave (PAW) method.[12,13] The cutoff kinetic energy of 500 eV is chosen for the plane wave basis. For the exchange-correlation energy, we employ the Perdew-Burke-Ernzerhof (PBE) functional with generalized gradient approximation (GGA).[14] Based on the test of convergence accuracy, we use (7 × 7 × 7) Monkhorst-Pack k-point mesh to represent the reciprocal space of the unit cell. By using the conjugated gradient algorithm, the crystal structure is fully optimized, the convergence criteria for energy and force are set to be 10^{-4} eV and 0.01 eV $Å^{-1}$, respectively. Furthermore, the climbing-image nudged elastic band (CI-NEB) method implemented in VASP was used to determine the diffusion energy barrier and the minimum energy pathways.[15]

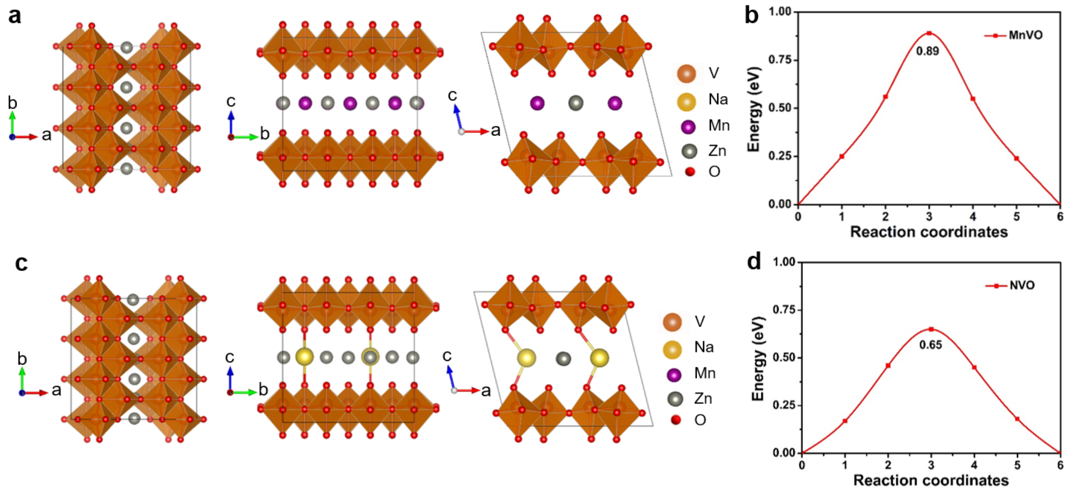

Figure S16. a) Possible migration pathways for Zn^{2+} in MnVO. b) Energy barriers along Zn^{2+}-migration pathways for MnVO c) Possible migration pathways for Zn^{2+} in NVO. d) Energy barriers along Zn^{2+}-migration pathways for NVO.

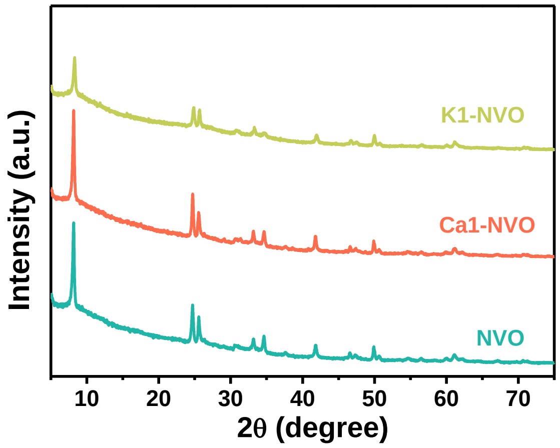

Figure S17. XRD patterns of as synthesized NVO, Ca1-NVO, and K1-NVO.

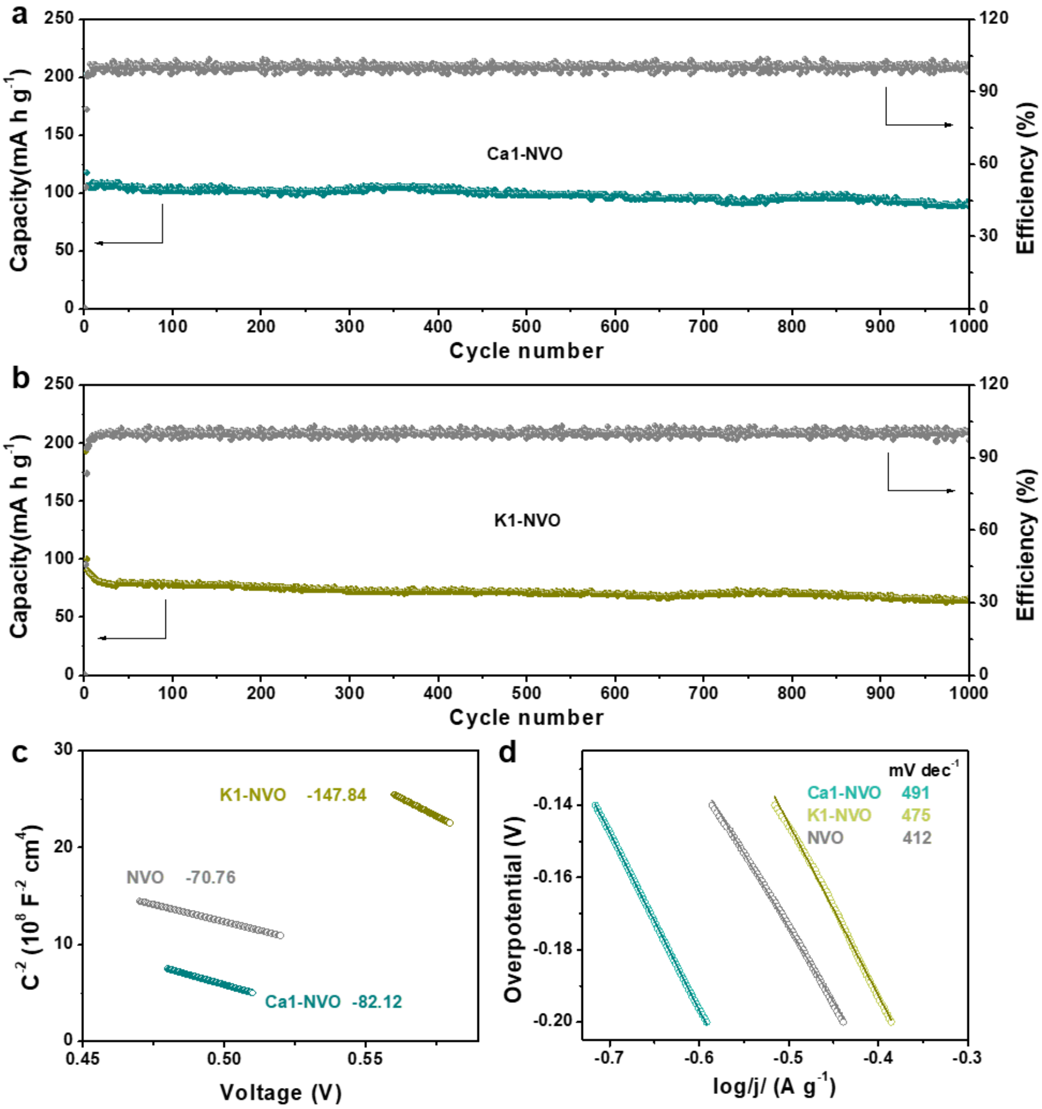

Figure S18. a) Long-term cycling performance of Ca1-NVO at a current rate of 4 A g^{-1}. b) Long-term cycling performance of K1-NVO at a current rate of 4 A g^{-1}. c) Mott-Schottky plots of NVO, Ca1-NVO, and K1-NVO. d) Tafel curves of NVO, Ca1-NVO, and K1-NVO at a sweep rate of 1 mV s^{-1}.

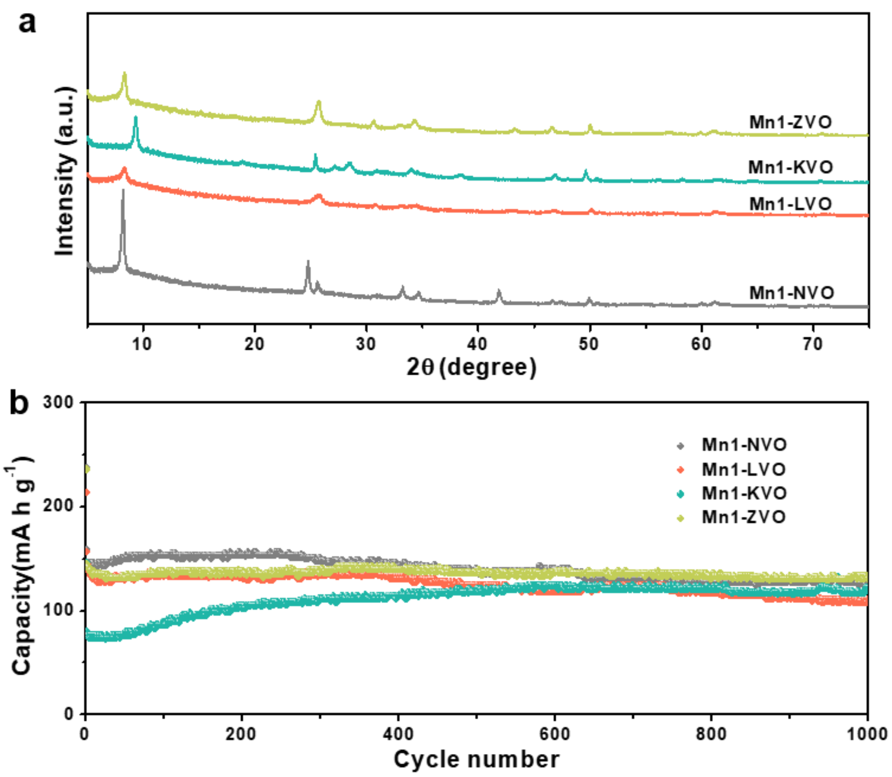

Figure S19. a) XRD patterns of as synthesized Mn1-NVO, Mn1-LVO, Mn1-KVO, and Mn1-ZVO. b) Long-term cycling performance of Mn1-NVO, Mn1-LVO, Mn1-KVO, and Mn1-ZVO at a current rate of 4 A g^{-1}.

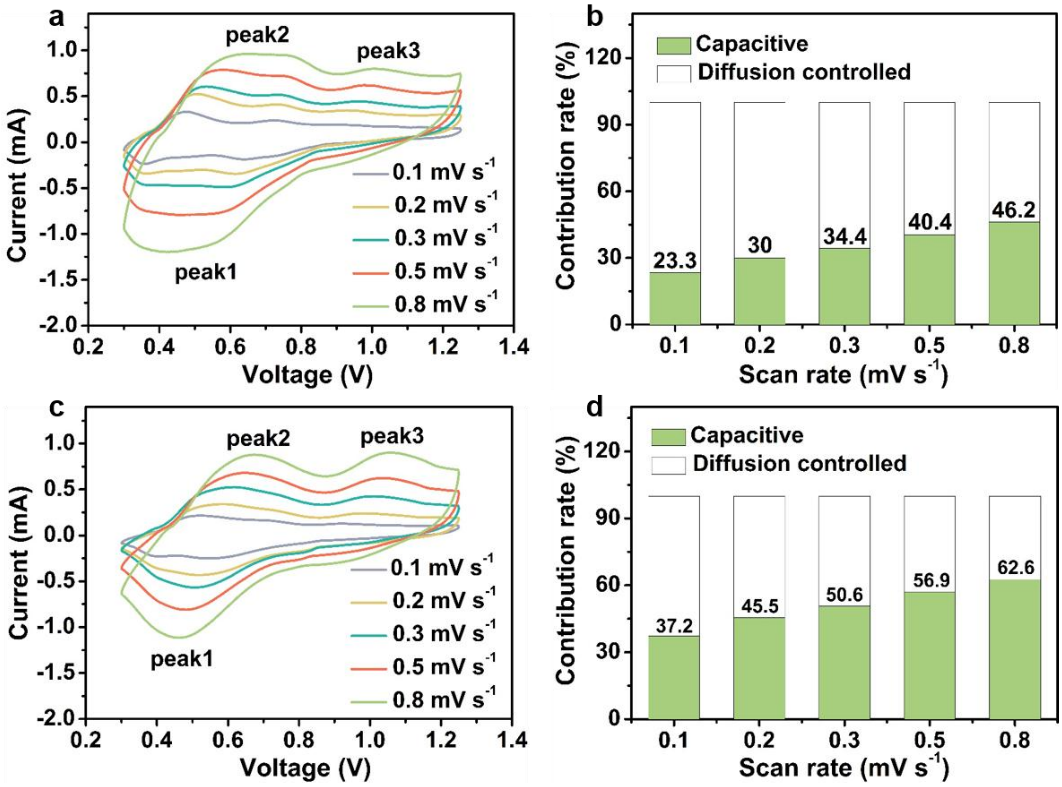

Figure S20. a) CV curves of NVO electrode at different scan rates. b) The capacitive contributions of NVO at scan rates of 0.1, 0.2, 0.3, 0.5, and 0.8 mV s^{-1}. c) CV curves of Mn2-NVO electrode at different scan rates. d) The capacitive contributions of Mn2-NVO at scan rates of 0.1, 0.2, 0.3, 0.5, and 0.8 mV s^{-1}.

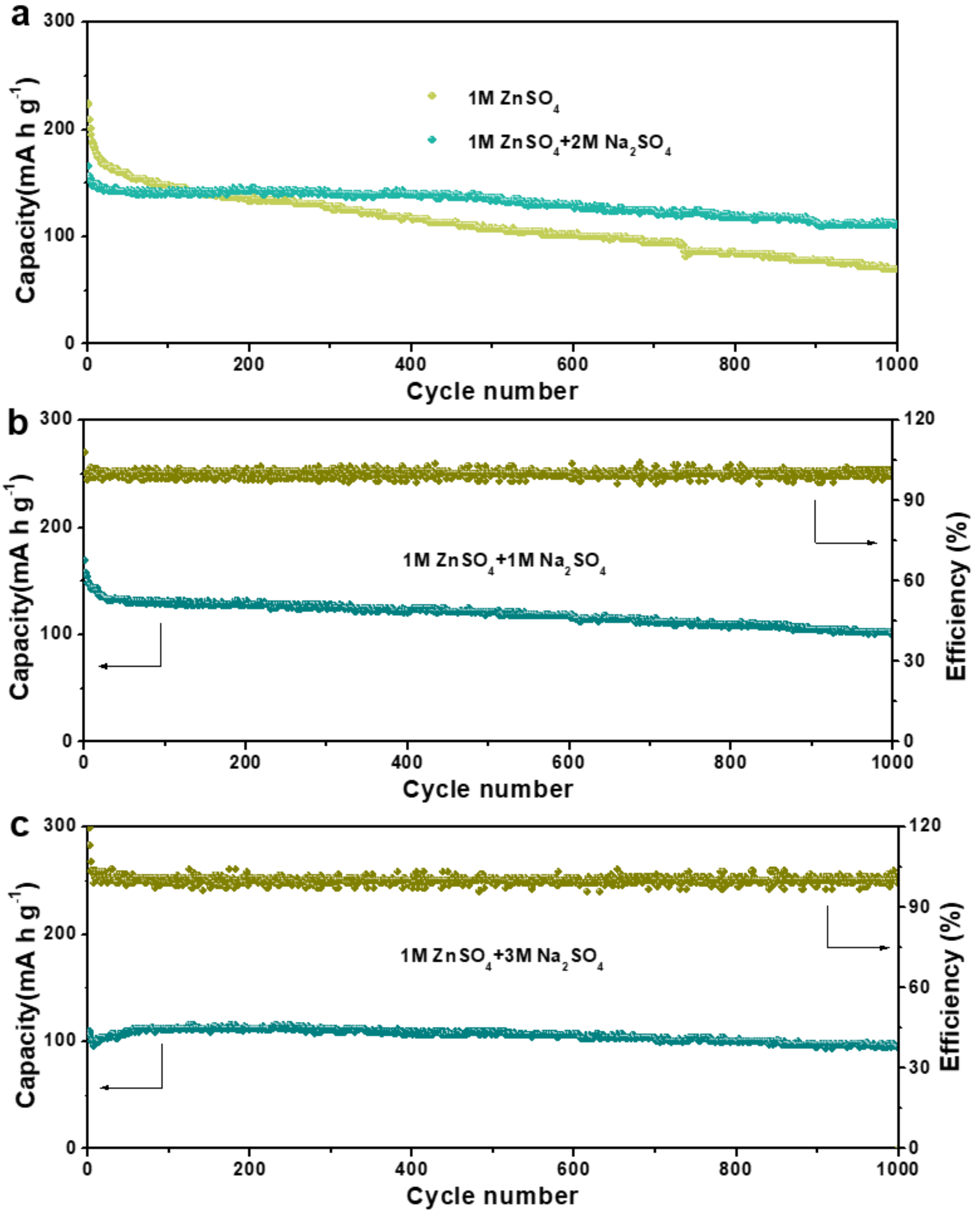

Figure S21. Cycling performance of Mn1-NVO in a) 1 M $ZnSO_4$ + 2 M Na_2SO_4, b) 1 M $ZnSO_4$ + 1 M Na_2SO_4 and c) 1 M $ZnSO_4$ + 3 M Na_2SO_4 electrolytes at 4 A g^{-1}. In 1 M $ZnSO_4$, the initial capacity of Mn1-NVO is 223 mA h g^{-1} and after 1000 cycles the capacity retention is 31%. When the concentration of Na_2SO_4 additives increases from 1 M to 3 M, the initial capacity of the Mn1-NVO is 168, 165 and 108 mA h g^{-1}, and the capacity retention is 59%, 68% and 87% after 1000 cycles, respectively. Therefore, increasing the concentration of Na_2SO_4 can suppress the dissolution of Mn1-NVO and show a high capacity retention, but the capacity of Mn1-NVO was decreased.

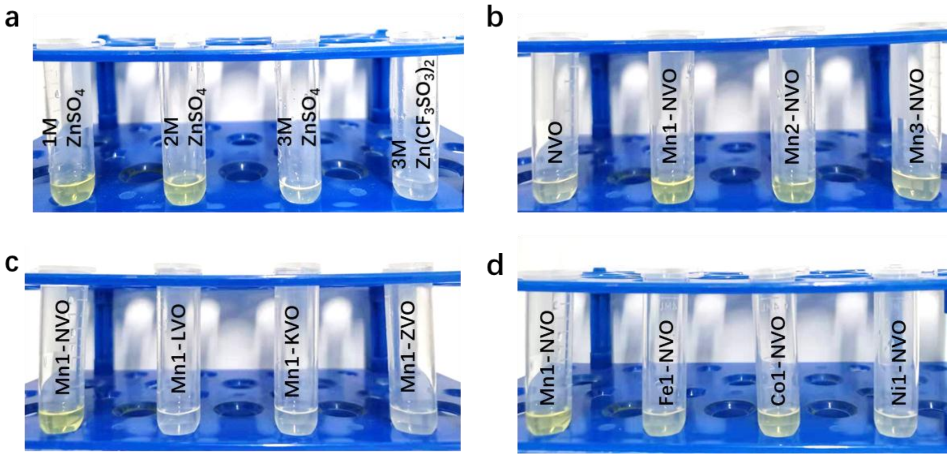

Figure S22. a) Optical images of Mn1-NVO electrodes immersed in 1M, 2M, 3M $ZnSO_4$, and 3M $Zn(CF_3SO_3)_2$ electrolytes for 12 h. b) Optical images of NVO, Mn1-NVO, Mn2-NVO and Mn3-NVO electrodes immersed in $ZnSO_4$ electrolytes for 12 h. c) Optical images of Mn1-NVO, Mn1-LVO, Mn1-KVO, and Mn1-ZVO electrodes immersed in 1M $ZnSO_4$ electrolyte for 12 h. d) Optical images of Mn1-NVO, Fe1-NVO, Co1-NVO, and Ni1-NVO electrodes immersed in 1M $ZnSO_4$ electrolyte for 12 h.

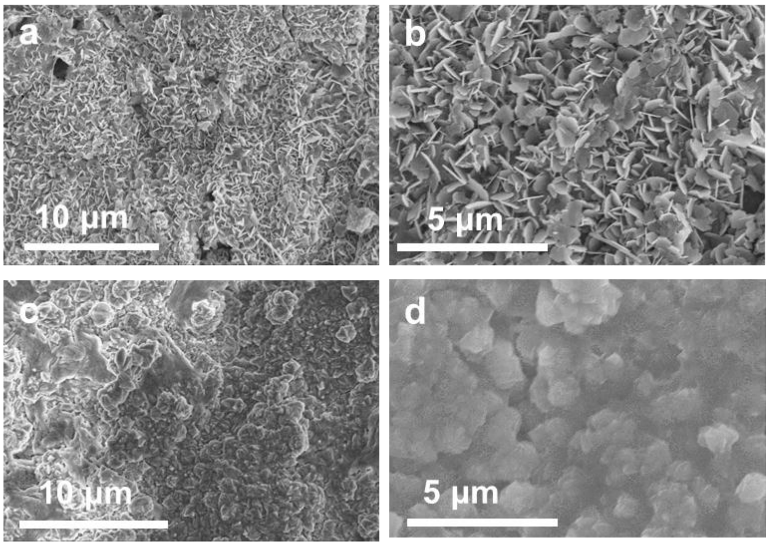

Figure S23. a,b) SEM images of Zn electrode surface from Zn//$ZnSO_4$//Mn1-NVO battery after 500 cycles at 4 A g^{-1}. c,d) SEM images of Zn electrode surface from Zn//Zn$(CF_3SO_3)_2$//Mn1-NVO battery after 500 cycles at 4 A g^{-1}.

Table S1. Molar ratio of elements in Mn1-NVO, Mn2-NVO, and Mn3-NVO

Sample	Na (mol)	Mn (mol)	V (mol)
Mn1-NVO	0.97	0.02	8
Mn2-NVO	0.33	0.65	8
Mn3-NVO	0.43	0.53	8

Table S2. Crystallographic data of the $(Na,Mn)V_8O_{20}$ powder obtained from Rietveld refinement

Space group *C2/m*	a=11.699(6)<br>b=3.647(8)<br>c=11.119(2)	α=90<br>β=104.05<br>γ=90	R_{wp}=0.0384<br>R_p=0.0224<br>R_{exp}=0.0092<br>chi^2=17.26	
Atom	x	z	U	Occupancy
Na1	0.061	0.506	0.02	0
Na2	0.568	0.478	0.02	0.1054
Mn1	0.061	0.506	0.02	0.6556
Mn2	0.568	0.478	0.02	0.2949
V1	0.8018	0.137	0.01	1
V2	0.0982	0.1378	0.01	1
O1	0.946	0.096	0.01	1
O2	0.849	0.283	0.01	1
O3	0.629	0.12	0.01	1
O4	0.274	0.1	0.01	1
O5	0.11	0.286	0.01	1

*Note: all atoms in 4i, and y = 0 for all atoms.

Table S3. Crystallographic data of the $Na(V,Mn)_8O_{20}$ powder obtained from Rietveld refinement

Space group *C2/m*	a=11.698(6)<br>b=3.648(8)<br>c=11.168(3)	α=90<br>β=104.87<br>γ=90	R_{wp}=0.0957<br>R_p=0.0475<br>R_{exp}=0.0093<br>chi^2=106.8	
Atom	x	z	U	Occupancy
Na1	0.061	0.506	0.02	0.125
Na2	0.568	0.478	0.02	0.125
Mn1	0.061	0.506	0.02	0.2409
Mn2	0.568	0.478	0.02	0.1003
V1	0.8018	0.137	0.01	0.8985
V2	0.0982	0.1378	0.01	0.8195
O1	0.946	0.096	0.01	1
O2	0.849	0.283	0.01	1
O3	0.629	0.12	0.01	1
O4	0.274	0.1	0.01	1
O5	0.11	0.286	0.01	1

*Note: all atoms in 4i, and y = 0 for all atoms.

Table S4. The mole ratio of Mn^{3+}:Mn^{2+} in Mn2-NVO and Mn3-NVO analysed from the XPS in Figure S1

Samples	Mn^{3+}	Mn^{2+}	Satellite
Mn2-NVO	1	0.2	0.55
Mn3-NVO	1	0.22	0.52

Table S5. The mole ratio of V^{5+}:V^{4+} in NVO, Mn1-NVO, Mn2-NVO, and Mn3-NVO analysed from the XPS in Figure S2

Samples	V^{5+}	V^{4+}
NVO	1	0.44
Mn1-NVO	1	0.47
Mn2-NVO	1	0.5
Mn3-NVO	1	0.53

Table S6. The molar ratio of V^{5+}:V^{4+}:V^{3+} in Mn1-NVO at the fully dischared and charged state

Sample	V^{3+}	V^{4+}	V^{5+}
Initial	-	0.32(516.2 eV)	0.68(517.5 eV)
Discharge-0.3V	0.18(515 eV)	0.46(516.3 eV)	0.36(517.5 eV)
Charge-1.25V	-	0.32(516.2 eV)	0.68(517.5 eV)

Table S7. The mole ratio of intercalated Zn^{2+} and absorbed Zn^{2+} in Mn1-NVO at the fully dischared and charged state

Sample	Intercalated Zn^{2+}	Absorbed Zn^{2+}
Discharge-0.3V	0.79(1022 eV)	0.21(1020.7 eV)
Charge-1.25V	0.38(1022 eV)	0.62(1020.7 eV)

Table S8. Comparison of various electrodes in aqueous ZIBs

Materials	Electrolyte	Capacity (mAh g^{-1}) at n A g^{-1}	Capacity retention after x cycle at y A g^{-1}	Ref
$(Na_{0.33},Mn_{0.65})V_8O_{20}\cdot nH_2O$	3M $Zn(CF_3SO_3)_2$	377(n=0.1)	99%(x=1000, y=4)	Our work
$(Zn,Mn)V_8O_{20}\cdot nH_2O$	3M $Zn(CF_3SO_3)_2$	359(n=0.1)	92%(x=1000, y=4)	Our work
$Zn_{0.25}V_2O_5\cdot nH_2O$	1M $ZnSO_4$	300 (n=0.05)	80% (x=1000, y=2.4)	[1]
$Ca_{0.25}V_2O_5\cdot nH_2O$	1M $ZnSO_4$	340(n= 0.2 C)	96% (x=3000, y=80C)	[2]
$NaV_3O_8\cdot 1.5H_2O$	1 M $ZnSO_4$ /1 M Na_2SO_4	380(n=0.1)	82%(x=1000, y=4)	[3]
$V_2O_5.nH_2O$	3M $Zn(CF_3SO_3)_2$	381 (n=0.06)	71% (x=900, y=6)	[4]
$H_2V_3O_8$	3M $Zn(CF_3SO_3)_2$	423 (n=0.1)	94.3% (x=1000, y=5)	[5]
$Na_2V_6O_{16}\cdot 1.63H_2O$	3M $Zn(CF_3SO_3)_2$	352 (n=0.05)	90% (x=6000, y=5)	[6]
LiV_3O_8	1M $ZnSO_4$	280 (n=0.016)	75% (x=65, y=0.133)	[7]
VS_2	1M $ZnSO_4$	190 (n=0.05)	98% (x=200, y=0.05)	[8]
$Zn_3V_2O_7(OH)_2\cdot 2H_2O$	1M $ZnSO_4$	213 (n=0.05)	68% (x=300, y=0.2)	[9]
$Na_{0.33}V_2O_5$	3M $Zn(CF_3SO_3)_2$	367.1(n=0.1)	93%(x=1000, y=1)	[10]
$Mg_{0.34}V_2O_5\cdot nH_2O$	3M $Zn(CF_3SO_3)_2$	353(n=0.1)	97%(x=2000, y=5)	[11]

Table S9. ICP-OES results of NVO and Mn2-NVO immersed in 1M $ZnSO_4$ electrolytes for 12 h

Samples	V (mol L^{-1})	Mn (mol L^{-1})	Na (mol L^{-1})
NVO	0.148	0	0.0068
Mn2-NVO	0.373	2.25	0.0053

References

[1] D. Kundu, B. D. Adams, V. Duffort, S. H. Vajargah, L. F. Nazar, *Nat. Energy* **2016**, 1, 16119.

[2] C. Xia, J. Guo, P. Li, X. Zhang, H. N. Alshareef, *Angew. Chem. Int. Ed.* **2018**, 57, 3943.

[3] F. Wan, L. Zhang, X. Dai, X. Wang, Z. Niu, J. Chen, *Nat. Commun.* **2018**, 9, 1656.

[4] M. Yan, P. He, Y. Chen, S. Wang, Q. Wei, K. Zhao, X. Xu, Q. An, Y. Shuang, Y. Shao, K. T. Mueller, L. Mai, J. Liu, J. Yang, *Adv. Mater.* **2018**, 30, 1703725.

[5] P. He, Y. Quan, X. Xu, M. Yan, W. Yang, Q. An, L. He, L. Mai, *Small* **2017**, 13, 1702551.

[6] P. Hu, T. Zhu, X. Wang, X. Wei, M. Yan, J. Li, W. Luo, W. Yang, W. Zhang, L. Zhou, Z. Zhou, L. Mai, *Nano Lett.* **2018**, 18, 1758.

[7] M. H. Alfaruqi, V. Mathew, J. Song, S. Kim, S. Islam, D. T. Pham, J. Jo, S. Kim, J. P. Baboo, Z. Xiu, K. Lee, Y. Sun, J. Kim, *Chem. Mater.* **2017**, 29, 1684.

[8] P. He, M. Yan, G. Zhang, R. Sun, L. Chen, Q. An, L. Mai, *Adv. Energy Mater.* **2017**, 1601920.

[9] C. Xia, J. Guo, Y. Lei, H. Liang, C. Zhao, H. N. Alshareef, *Adv. Mater.* **2018**, 30, 1705580.

[10] P. He, G. Zhang, X. Liao, M. Yan, X. Xu, Q. An, J. Liu, L. Mai, *Adv. Energy Mater.* **2018**, 8, 1702463.

[11] F. Ming, H. Liang, Y. Lei, S. Kandambeth, M. Eddaoudi, H. N. Alshareef, *ACS Energy Lett.* **2018**, 3, 2602.

[12] G. Kresse, J. Furthmüller, *Phys. rev. B* **1996,** *54*, 11169.

[13] G. Kresse, D. Joubert, *Phys. Rev. B* **1999,** *59*, 1758.

[14] J. P. Perdew, K. Burke, M. Ernzerhof, *Phys. Rev. Lett.* **1996,** *77*, 3865.

[15] G. Henkelman, B. P. Uberuaga, H. Jónsson, *J. Chem. Phys.* **2000,** *113*, 9901-9904.
